# Metabolome profiling and transcriptome analysis unveiling the crucial role of magnesium transport system for magnesium homeostasis in tea plants

**DOI:** 10.1093/hr/uhae152

**Published:** 2024-06-03

**Authors:** Jing Li, Ting Wen, Ruiming Zhang, Xinlong Hu, Fei Guo, Hua Zhao, Pu Wang, Yu Wang, Dejiang Ni, Mingle Wang

**Affiliations:** National Key Laboratory for Germplasm Innovation and Utilization of Horticultural Crops, College of Horticulture and Forestry Sciences, Huazhong Agricultural University, Wuhan 430070, China; Joint International Research Laboratory of Germplasm Innovation and Utilization of Horticultural Crops, Huazhong Agricultural University, Wuhan 430070, China; National Key Laboratory for Germplasm Innovation and Utilization of Horticultural Crops, College of Horticulture and Forestry Sciences, Huazhong Agricultural University, Wuhan 430070, China; Joint International Research Laboratory of Germplasm Innovation and Utilization of Horticultural Crops, Huazhong Agricultural University, Wuhan 430070, China; National Key Laboratory for Germplasm Innovation and Utilization of Horticultural Crops, College of Horticulture and Forestry Sciences, Huazhong Agricultural University, Wuhan 430070, China; Joint International Research Laboratory of Germplasm Innovation and Utilization of Horticultural Crops, Huazhong Agricultural University, Wuhan 430070, China; National Key Laboratory for Germplasm Innovation and Utilization of Horticultural Crops, College of Horticulture and Forestry Sciences, Huazhong Agricultural University, Wuhan 430070, China; Joint International Research Laboratory of Germplasm Innovation and Utilization of Horticultural Crops, Huazhong Agricultural University, Wuhan 430070, China; National Key Laboratory for Germplasm Innovation and Utilization of Horticultural Crops, College of Horticulture and Forestry Sciences, Huazhong Agricultural University, Wuhan 430070, China; Joint International Research Laboratory of Germplasm Innovation and Utilization of Horticultural Crops, Huazhong Agricultural University, Wuhan 430070, China; National Key Laboratory for Germplasm Innovation and Utilization of Horticultural Crops, College of Horticulture and Forestry Sciences, Huazhong Agricultural University, Wuhan 430070, China; Joint International Research Laboratory of Germplasm Innovation and Utilization of Horticultural Crops, Huazhong Agricultural University, Wuhan 430070, China; National Key Laboratory for Germplasm Innovation and Utilization of Horticultural Crops, College of Horticulture and Forestry Sciences, Huazhong Agricultural University, Wuhan 430070, China; Joint International Research Laboratory of Germplasm Innovation and Utilization of Horticultural Crops, Huazhong Agricultural University, Wuhan 430070, China; National Key Laboratory for Germplasm Innovation and Utilization of Horticultural Crops, College of Horticulture and Forestry Sciences, Huazhong Agricultural University, Wuhan 430070, China; Joint International Research Laboratory of Germplasm Innovation and Utilization of Horticultural Crops, Huazhong Agricultural University, Wuhan 430070, China; National Key Laboratory for Germplasm Innovation and Utilization of Horticultural Crops, College of Horticulture and Forestry Sciences, Huazhong Agricultural University, Wuhan 430070, China; Joint International Research Laboratory of Germplasm Innovation and Utilization of Horticultural Crops, Huazhong Agricultural University, Wuhan 430070, China; National Key Laboratory for Germplasm Innovation and Utilization of Horticultural Crops, College of Horticulture and Forestry Sciences, Huazhong Agricultural University, Wuhan 430070, China; Joint International Research Laboratory of Germplasm Innovation and Utilization of Horticultural Crops, Huazhong Agricultural University, Wuhan 430070, China

## Abstract

Magnesium (Mg^2+^) is a crucial nutrient for the growth and development of *Camellia sinensis* and is closely related to the quality of tea. However, the underlying mechanisms responding to low-Mg ^2+^ stress in tea plants remain largely unknown. In this study, photosynthetic parameters, metabolomics, and transcriptomics were utilized to explore the potential effects of low Mg^2+^ on the growth and metabolism of *C. sinensis*. Low-Mg^2+^ treatment increased the ratio of shoot dry weight to root dry weight but decreased the photosynthesis of *C. sinensis*. Forty and thirty metabolites were impacted by Mg^2+^ shortage in *C. sinensis* shoots and roots, respectively. Integrated transcriptome and metabolome analyses revealed the possible reasons for the decreased contents of chlorophyll and catechins and the increased theanine content in *C. sinensis* roots. Weighted gene co-expression network analysis indicated that the Mg^2+^ transport system was essential in the regulation of Mg^2+^ homeostasis in *C. sinensis*, in which *CsMGT5* was identified to be the key regulator according to *CsMGT5*-overexpressing and complementary assays in *Arabidopsis thaliana*. Moreover, silencing of *CsMGT5 in vivo* reduced the content of chlorophyll in *C. sinensis* shoots. In addition, *CsMGT5* might collaborate with ammonium transporters to keep the amino acid content steady, suggesting its potential application for tea quality improvement. All these findings demonstrate the key roles of *CsMGT*s for Mg^2+^ homeostasis in *C. sinensis*, providing a theoretical basis for Mg^2+^ efficient utilization in plants.

## Introduction

The tea plant is a perennial evergreen plant that originated in China. It possesses multiple health benefits and an important economic value due to the high abundance of secondary metabolites, mainly including flavonoids, theanine, and caffeine [1]. Tea taste, aroma, and leaf color are three of the most crucial indicators for evaluating tea quality [2]. Bitter, astringent, fresh, and sweet are considered the main attributes of tea taste [3]. Catechins and anthocyanins possess bitter and astringent properties, while caffeine is mainly associated with bitter taste [4]. Theanine is regarded as the flavoring substance of tea liquor, and has a key role in assessing the taste as fresh and brisk [5]. The sweet substances mainly comprise sweet amino acids, soluble sugars, and some organic acids [6]. The aroma type is the comprehensive effect of many aroma components, and is determined by a range of chemical constituents, chiefly including alcohols, aldehydes, ketones, acids, esters, lactones, phenols, oxygen heterocyclics, sulfur-containing compounds, and nitrogen (N)-containing compounds [7]. Leaf color mainly depends on the change in relative contents of chlorophyll (Chl), carotenoid, and anthocyanin in leaves [8]. Environmental factors associated with the quality of *Camellia sinensis* have been widely studied. Drought suppressed the metabolisms of indole-3-acetic acid (IAA), abscisic acid (ABA), gibberellin (GA3), and brassinosteroid (BR) by affecting the expression patterns of related genes in tea plant shoots [9]. Furthermore, drought stress resulted in obvious decreases in catechins, caffeine, theanine, and some free amino acids in tea plant leaves, which were involved in the different expression of genes regulating these secondary metabolites [10]. To adapt to cold stress, cell wall modification and enhanced starch metabolism conferred freezing tolerance in *C. sinensis* [11]. Moreover, the MAPK-dependent ethylene and ICE-CBF-COR signaling pathways are the two primary mechanisms of the response to cold shock in *C. sinensis* [12]. Heat stress decreased the anthocyanin content in tea leaves, and nine HSPs (heat shock proteins) and one HSF (heat shock transcription factor) might be involved in the high-temperature response in *C. sinensis* [13]. Furthermore, light quality contributes to the changes of leaf color. Red light favored the accumulation of flavonoid and Chl, and blue light facilitated the metabolism of carotenoid [14]. Besides, shading promoted the accumulation of Chl, whereas the contents of some catechins showed a decline in tea buds [15].

Nutrients not only have an irreplaceable role in the growth of *C. sinensis*, but are also closely related to the quality and yield of *C. sinensis*. N, phosphorus (P) and potassium (K) shortage affected the metabolism of the three most important substances (i.e. catechin, theanine, and caffeine) in the tea plant, which may be due to shared responsive transcription factors (TFs) and intermediate factors [16]. Moreover, our laboratory found that N starvation promoted amino acid accumulation but attenuated the metabolism of polyphenols and caffeine. Interestingly, flavonoid metabolism-related genes remained active under N starvation, suggesting that flavonoid metabolism might be a crucial pathway in the response to N deficiency [17]. P shortage debased tea quality by reducing the contents of total polyphenols, flavonoids, and total free amino acids [18]. Insufficient K could threaten the growth of the tea plant, disturb the synthesis of sugars and proteins, and affect the contents of quality components [18, 19]. Conversely, adequate K nutrition prompted the biosynthesis of aroma components and some free amino acids, and enhanced resistance to adversity by activating different enzymes, thus promoting the yield and quality of *C. sinensis* [6, 20, 21]. Exogenous application of calcium enhanced the drought tolerance of tea plants by affecting the galactinol biosynthesis pathway and mediating the regulation of the stomatal aperture in guard cells [22], and was capable of adapting to high temperature by modulating the expression patterns of 45 key genes [23]. Mg^2+^ is a indispensable nutrient to maintain the productivity and quality of crops by participating in multiple biochemical processes [24, 25]. Mg^2+^ deficiency is a common problem in crop production, especially for crops cultivated in lateritic and leached acid soils [26]. Therefore, it is necessary to elucidate the mechanism of Mg^2+^ deficiency in plants. Mg^2+^ deficit led to a decline in CO_2_ assimilation, whereas the contents of starch and sucrose were increased in *Citrus sinensis* leaves [27]. Further transcriptome analysis indicated that signal transduction, protein phosphorylation, and carbohydrate accumulation might enhance tolerance to Mg^2+^ deficiency in citrus [27]. The ratio of shoot/root dry weight increased in *Coffea arabica* under Mg^2+^ starvation, possibly due to the accumulation of carbohydrates in leaves [28]. Moreover, Mg^2+^ depletion resulted in chlorosis in banana leaves, but these leaves could regain their green color if Mg^2+^ was resupplied [29]. The content of Chl and photosynthetic efficiency were likely suppressed in *Saccharum* spp. leaves under Mg^2+^ shortage conditions, but the lignin content was increased in roots, an effect that might be involved in the differential expression of several Mg^2+^ transporters (MGTs) and TFs (TCPs, NACs, GATAs, and ERFs) [30]. In plants, MGTs and Mg^2+^/H^+^ exchanger (MHX) are two important Mg^2+^ transporting systems and are favorable for adapting to fluctuations of Mg^2+^concentration; the former have been widely investigated [31, 32]. In *Arabidopsis*, a total of 10 MGT members have been identified, of which 9 have been confirmed to possess Mg^2+^ transport capacity in yeast *mrs2* mutant complementarity experiments [33]. Nine genes were identified as *OsMGT*s in rice, and four of them exhibited Mg^2+^ transport capacity with the help of yeast strain CM66 [34]. There are 12 MGT members in maize and 6 of them were proved to mediate Mg^2+^ uptake using a complementation assay in *Salmonella* mutant MM281 [35]. Eight MGT members have been reported in citrus, and *PtrMGT5* was verified to promote resistance to Mg^2+^ deficiency stress by regulating Mg^2+^ balance [36]. Furthermore, some MGT members were involved in more physiological processes. For example, overexpression of *AtMGT1* could ameliorate aluminum toxicity [37], and ZmMGT-mediated Mg^2+^ accumulation in maize roots enhanced aluminum resistance [38]. AtMGT2 and AtMGT3 maintained Mg^2+^ homeostasis to adapt to low-calcium conditions [39]. PbrMGT7 was involved in pear pollen development [40]. Besides, AtMGT10 was necessary for chloroplast development, and mutation of *MGT10* resulted in the yellow reticulated-leaf vein phenotype [41].

Tea plantations are mostly distributed in red soil areas with strong weathering and leaching, which causes the loss of Mg^2+^ due to the weak soil adsorption capacity [42, 43]. Moreover, biased application of N fertilizer has further aggravated the imbalance of Mg^2+^ in tea plantations [44]. Therefore, tea plants are generally confronted with the crisis of Mg^2+^ limitation. In recent years, botanists have paid attention to this issue and tried to uncover the relationship between tea plants and Mg^2+^. Under hydroponic conditions, Mg^2+^ deficiency decreased the contents of polyphenols, free amino acids, caffeine, and Chl in *C. sinensis* shoots [45]. On the contrary, the application of Mg^2+^ fertilizer contributed to the tea yield and reduced the ratio of total polyphenols to amino acids in tea leaves, which is possibly due to the enhanced N assimilation under the regulation of *glutamine synthetase 1.1* (*CsGS1.1*) [43, 46, 47]. Previously, 10 *CsMGT*s were identified from tea plants by our laboratory, and CsMGT5 was confirmed to participate in the absorption of Mg^2+^ under limited Mg^2+^ concentrations [48]. Besides, CsMGT10 played a crucial role in leaf vein greening [49]. However, the underlying regulatory mechanisms between Mg^2+^ and growth and quality of *C. sinensis* remain largely unknown and need further exploration. In this study, we attempt to explore the response of tea plants to the Mg^2+^ deficiency and clarify the role of the CsMGT system under low Mg^2+^ availability. Firstly, photosynthetic physiological characteristic parameters were measured to uncover the relationship between Mg^2+^ deficiency and tea plant growth. Then, metabolomic analysis combined with RNA-seq was utilized to reveal the global change in metabolites and key genes in *C. sinensis* shoots and roots. Furthermore, heterologous stable expression and antisense oligodeoxynucleotide (asODN) techniques were employed to clarify the function of CsMGT5 in transgenic *Arabidopsis thaliana* and *in vivo*, respectively. Finally, a proposed model is summarized to illustrate the potential regulation network of *C. sinensis* in response to low-Mg^2+^ stress. These findings help to deepen our awareness of the relationship between the Mg^2+^ transport system and tea plant growth and tea fresh leaf quality, and also lay a foundation for further exploration of regulatory mechanisms of Mg^2+^ nutrition in plants.

## Results

### Change patterns of physiological indices and photosynthetic characteristic parameters under Mg^2+^-deficient conditions

**Figure 1:**
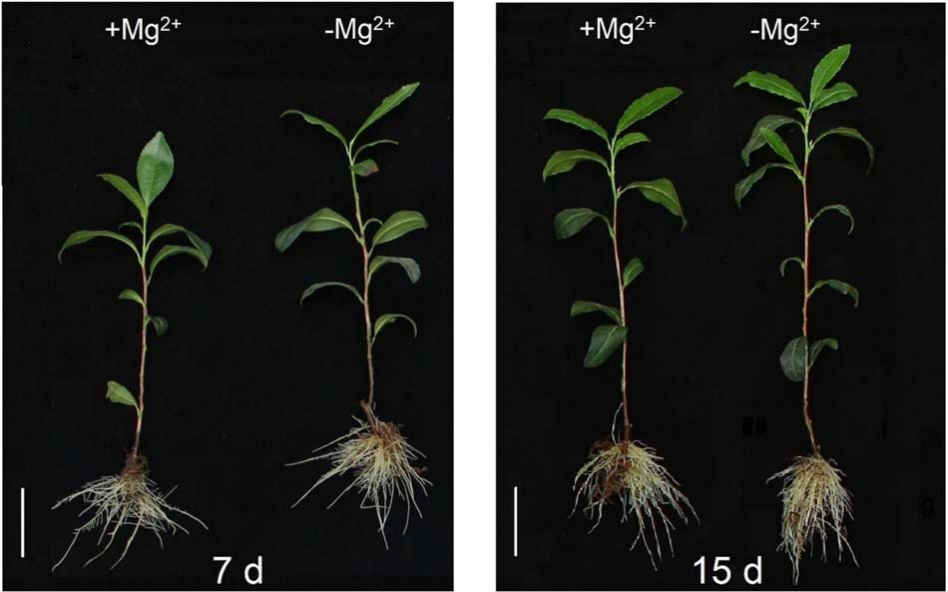
Phenotypes of representative tea plants under Mg^2+^-sufficient (+Mg^2+^) and Mg^2+^-deficient (-Mg^2+^) conditions for 7 and 15 days. Scale bar = 2 cm.

When exposed to Mg^2+^-deficient conditions, the dry weight of tea plant roots did not present a significant change (Fig. 1 and Supplementary Data Table S1). On the contrary, the dry weight of tea shoots showed an upward trend (Fig. 1 and Supplementary Data Table S1). Furthermore, tea plants under Mg^2+^-deficient conditions possessed a higher dry weight of shoots/roots than tea plants under Mg^2+^-sufficient conditions (Supplementary Data Fig. S1).

**Figure 2:**
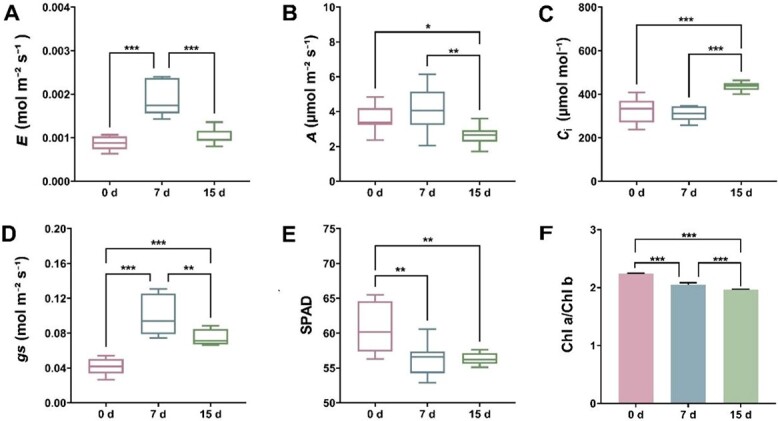
Patterns of photosynthetic physiological characteristic parameters in *C. sinensis*. **A** Transpiration rate (*E*). **B** Net photosynthetic rate (*A*). **C** Intercellular CO_2_ concentration (*C*_i_). **D** Stomatal conductance (*g*_s_). **E** SPAD value. There were 10 biological replicates for each treatment. **F** Ratio of Chl a to Chl b, *n* = 4. ‘0 d’ indicates seedlings before low-Mg^2+^ treatment. ‘7 d’ and ‘15 d’ represent seedlings treated with low-Mg^2+^ treatment for 7 and 15 days, respectively. The data represent mean ± standard deviation. *P < 0.05; **P < 0.01; ***P < 0.001 (Student’s *t*-test).

Photosynthesis is a critical biological process for green plants by providing nutrients and accumulating energy for growth and development, and a series of physiological characteristic parameters including net photosynthetic rate (*A*), transpiration rate (*E*), intercellular CO_2_ concentration (*C*_i_), and stomatal conductance (*g*_s_) are vital factors to measure photosynthetic intensity [50]. After low-Mg^2+^ treatments, *E* and *g*_s_ presented an initial increase followed by a decrease (Fig. 2A and D). Additionally, *A* showed a downward trend while *C*_i_ displayed an opposite change tendency after 7 days of low-Mg^2+^ treatments (Fig. 2B and C). The content of Chl has a direct impact on the photosynthetic efficiency of plants, and the determination of the SPAD value can effectively reflect the Chl content of leaves in plants. Under Mg^2+^-deficient conditions, the SPAD readings showed a general decline (Fig. 2E). Furthermore, low Mg^2+^ led to a reduction in the ratio of Chl a to Chl b (Fig. 2F). Interestingly, *A* did not change obviously after the transition to the low-Mg^2+^ conditions for 7 days, while *A* exhibited a downtrend when treated with low Mg^2+^ for 15 days (Fig. 2B). There are two possible reasons accounting for the variation. Initially, long-term Mg^2+^ deficiency might damage photosynthetic organs, which could affect carbon (C) fixation. Additionally, long-term Mg^2+^ deficiency probably influenced the synthesis of Chl. Collectively, these findings suggested that Mg^2+^ played a crucial part in the growth of *C. sinensis*.

### Global metabolic profiling of *C. sinensis* under Mg^2+^-deficient conditions

**Figure 3:**
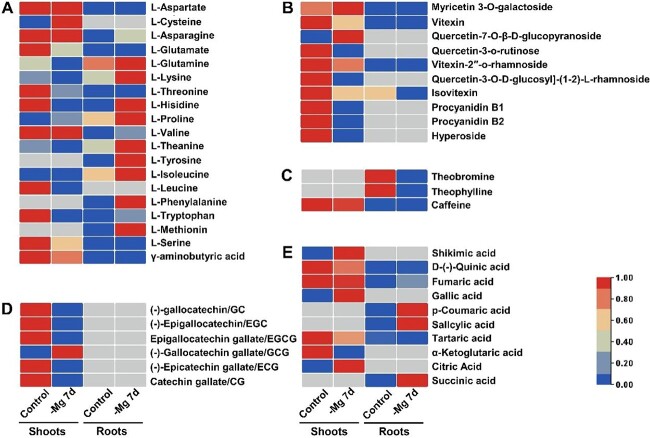
Metabolomic profiles in *C. sinensis* under Mg^2+^ deficiency. Contents of (**A**) free amino acids, (**B**) flavonoids, (**C**) alkaloids, (**D**) catechins and (**E**) organic acids in *C. sinensis* shoots and roots after 7 days of low-Mg^2+^ treatment. The contents of each kind of metabolite were analyzed with row standardization. The gray block indicates that the corresponding substance was undetectable.

To further explore the impact of Mg^2+^ deficiency on the metabolism of *C. sinensis*, metabonomic analysis was carried out based on the metabolic spectrum library built by our laboratory. In shoots, a total of 40 metabolites were identified, including 16 free amino acids, 10 flavonoids, caffeine, 6 catechins, and 7 organic acids (Fig. 3 and Supplementary Data Table S2). The contents of 12 amino acids showed a downward trend under low-Mg^2+^ stress (Fig. 3A). It is noteworthy that the content of theanine, which is closely related to the taste and aroma of tea, decreased obviously (Fig. 3A). Moreover, the content of caffeine decreased, whereas the contents of catechins, flavonoids, and organic acids were affected to varying degrees (Fig. 3B–E).

In roots, a total of 30 metabolites, comprising 17 amino acids, 4 flavonoids, 3 alkaloids, and 6 organic acids, were detected (Fig. 3 and Supplementary Data Table S2). The contents of γ-aminobutyric acid (GABA) decreased, whereas the contents of other identified amino acids increased (Fig. 3A). Furthermore, the content of theanine in roots increased obviously, which was completely opposite to the trend in shoots (Fig. 3A). Besides, the contents of alkaloids exhibited a falling trend, while low-Mg^2+^ treatment increased the contents of most organic acids (Fig. 3C and E). Taken together, these results suggested that low-Mg^2+^ treatments affected the biosynthesis of metabolites in *C. sinensis*.

### RNA-seq and differentially expressed gene annotation

To probe into the molecular mechanisms of Mg^2+^ deficiency on the metabolic profiles of tea plants, transcriptome analyses were performed, and each sample generated ~6.49 Gb of data. After filtering the raw data, the average valid data of each sample was 6.42 Gb. Each of the transcriptomes had 44.5–45.5% GC content, with an average GC content of 45.3%. The Q20 values and Q30 values of eight samples were 99.59–99.70 and 95.54–96.14%, respectively. To further validate the quality of the transcriptome assembly, transcriptomic unigenes showed a high mapping rate of 92.45% into the *C. sinensis* reference genome.

To reveal the dynamics of gene expression in tea plants under Mg^2+^ deficiency (i.e, –Mg 7 d), two comparisons (i.e. –Mg 7 d_shoots vs control_shoots and –Mg 7 d_roots *vs* control_roots) were evaluated and a total of 4232 differentially expressed genes (DEGs) were identified. Specifically, 2522 DEGs (1277 upregulated and 1245 downregulated) and 1710 DEGs (1092 upregulated and 618 downregulated) were identified between –Mg 7 d and control in tea plant shoots and roots, respectively.

**Figure 4:**
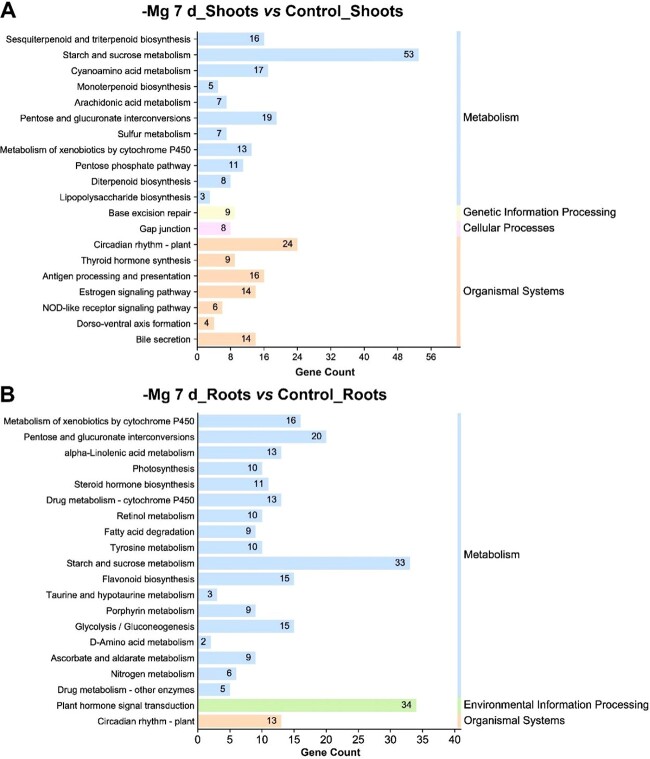
Top 20 KEGG pathways of enrichment of DEGs in comparisons (**A**) -Mg 7 d_shoots vs control_shoots and (**B**) -Mg 7 d_roots vs control_roots.

To fully comprehend the function of DEGs, GO enrichment and KEGG pathway analyses were performed. According to GO analysis, the significantly enriched terms in tea plant shoots were associated with ‘protein folding’, ‘structural constituent of cytoskeleton’, and ‘phosphatase activity’ (Supplementary Data Fig. S2A), while ‘oxidation–reduction process’, ‘extracellular region’, and ‘transporter activity’ were significantly enriched in tea plant roots (Supplementary Data Fig. S2B). According to the KEGG database, the top 20 DEGs in tea plant shoots were divided into four categories, comprising ‘metabolism’, ‘genetic information processing’, ‘cellular processes’, and ‘organismal systems’ (Fig. 4A), and the top 20 DEGs in tea plant roots were assigned to three groups: ‘metabolism’, ‘environmental information processing’, and ‘organismal systems’ (Fig. 4B). Obviously, Mg^2+^ deficiency mainly impacted the metabolism of tea plants. Further analysis found that ‘starch and sucrose metabolism’ and ‘circadian rhythm’ in tea plant shoots and roots changed greatly (Fig. 4), which may be an adaptation to Mg^2+^ shortage. Besides, Mg^2+^ shortage affected the biosynthesis of terpenoids in relation to aroma in shoots (Fig. 4A). Furthermore, the number of DEGs accumulated in ‘plant hormone signal transduction’ was the largest in roots (Fig. 4B), which might contribute to the regulation of growth and development and confer tolerance to Mg^2+^-deficiency stress in tea plants. In addition, Mg^2+^ deprivation could affect photosynthesis, N metabolism, flavonoid biosynthesis, and other vital processes, which was consistent with the metabolome outcome.

### Transcription-level regulation of chlorophyll metabolism in *C. sinensis* shoots under Mg^2+^ deficiency

**Figure 5:**
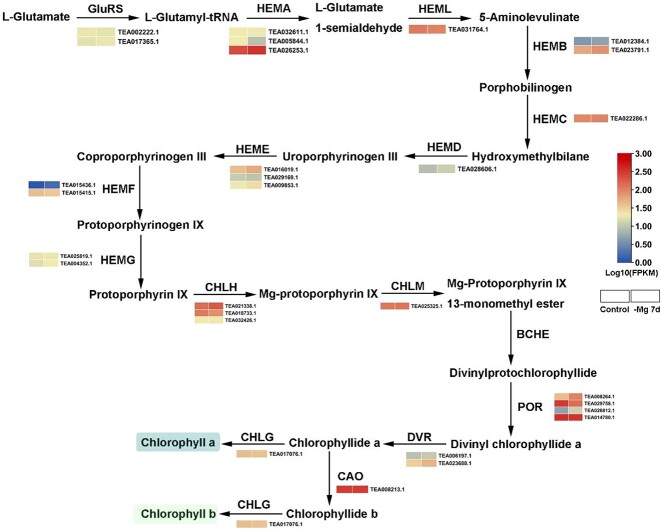
Expression of key genes related to the Chl biosynthesis pathway in *C. sinensis* shoots under Mg^2+^ deficiency. The color scale bar displays the normalized FPKM (log_10_-transformed fold-changes); red represents high expression and blue denotes low expression.

To clarify the molecular mechanisms by which Mg^2+^ deficiency decreased the Chl content, dynamic changes in gene expression were summarized in the Chl metabolism pathway based on the transcriptome profiles (Fig. 5 and Supplementary Data Table S3). Overall, 29 unigenes, which encode 15 enzymes, were identified by mapping to KEGG. In the conversion reaction of l-glutamate to 5-aminolevulinate, the majority of genes encoding glutamyl-tRNA synthetase (GluRS), glutamyl-tRNA reductase (HEMA), and glutamate-1-semialdehyde aminotransferase (HEML) were downregulated. However, genes encoding porphobilinogen synthase (HEMB), hydroxymethylbilane synthase (HEMC), uroporphyrinogen-III synthase (HEMD), uroporphyrinogen decarboxylase (HEME), coproporphyrinogen III oxidase (HEMF), menaquinone-dependent protoporphyrinogen oxidase (HEMG), Mg^2+^ chelatase subunit H (CHLH), and Mg^2+^-protoporphyrin *O*-methyltransferase (CHLM), which participated in the process from 5-aminolevulinate to Mg-protoporphyrin IX 13-monomethyl ester, were positively regulated by Mg^2+^-limited conditions. Protochlorophyllide reductases (PORs) were reported to catalyze the reduction of divinylprotochlorophyllide to divinyl chlorophyllide a, and played a vital role in photomorphogenesis. The expression profiles of *POR*s exhibited a complex trend under Mg^2+^-limited conditions, in which the expression levels of two transcripts (i.e. TEA008264.1 and TEA026812.1) increased and the other two transcripts (i.e. TEA029758.1 and TEA014780.1) decreased. Divinyl chlorophyllide a 8-vinyl-reductase (DVR) was responsible for the synthesis of chlorophyllide a, whose expression was induced by low-Mg^2+^ treatment. *CAO* was the key gene that catalyzed the biosynthesis of Chl b and directly affected the content of Chl b, whereas its expression was repressed by Mg^2+^ starvation. CHLG was considered to catalyze the formation of Chl a/b from chlorophyllide a/b and participate in the feedback control of the whole Chl biosynthesis pathway, and its expression pattern was inhibited by low-Mg^2+^ treatments. Hence, we speculated that the downregulated expression of key genes, including *CAO* and *CHLG*, may lead to the reduction of Chl concentrations in *C. sinensis* shoots.

### Transcription-level regulation of theanine metabolism in *C. sinensis* under Mg^2+^ deficiency

**Figure 6:**
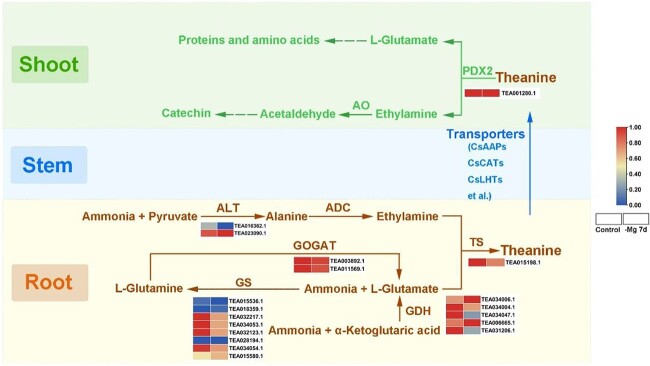
Expression of key genes related to the theanine metabolic pathway in *C. sinensis* under Mg^2+^ deficiency. The expression profiles of each gene were analyzed with row standardization. Red and blue bars denote high expression and low expression, respectively.

Metabolomic analysis showed that the content of theanine was obviously affected by low-Mg^2+^ treatment in both tea plant shoots and roots (Fig. 3). To reveal the underlying mechanisms of the change patterns, five genes [glutamate dehydrogenase (*GDH*), *GS*, glutamine-2-oxoglutarate synthetase gene (*GOGAT*), alanine amino transferase (*ALT*), and theanine synthetase (*TS*)] related to theanine synthesis and one gene [pyridoxine biosynthesis 2 (*PDX2*)] involved in theanine hydrolysis were identified from roots and shoots, respectively (Fig. 6 and Supplementary Data Table S4). Specifically, there are two pathways to synthesize l-glutamate: the glutamate GDH pathway and the GS/GOGAT pathway. Obviously, most of the genes encoding GDH, GS, and GOGAT in these two pathways were suppressed by low-Mg^2+^ stress. Meanwhile, ALT catalyzed the formation of alanine from ammonia and pyruvate, followed by catalysis of arginine decarboxylase (ADC) to form ethylamine. Theanine was synthesized from l-glutamate and ethylamine, catalyzed by TS, whose expression level was significantly inhibited by Mg^2+^ deprivation. Subsequently, theanine was transported from roots to shoots through the xylem by a series of transport proteins (AAPs, CATs, and LHTs). In shoots, PDX2 was considered to hydrolyze theanine to l-glutamate and ethylamine, while the expression was slightly upregulated by low-Mg^2+^ stress. Finally, l-glutamate was converted into protein and other amino acids, and ethylamine was transformed into catechins. Collectively, the significant change in theanine content and the expression of encoding genes under Mg^2+^ deprivation suggested that Mg^2+^ was instrumental in theanine metabolism.

### Transcription-level regulation of catechin metabolism in *C. sinensis* under Mg^2+^ deficiency

**Figure 7:**
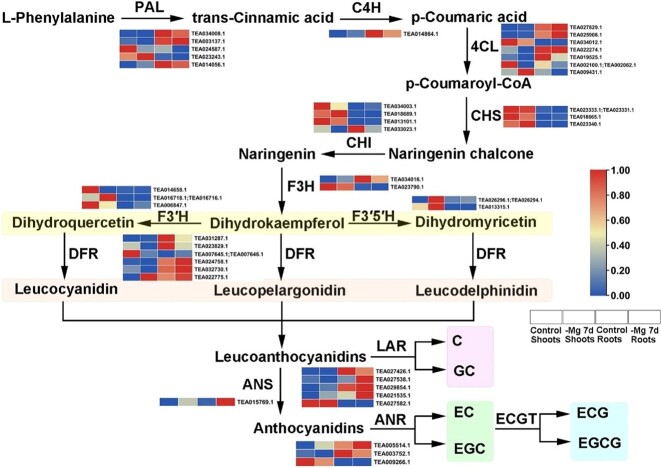
Expression of key genes related to the catechin biosynthesis pathway in *C. sinensis* under Mg^2+^ deficiency. Expression profiles of each gene were analyzed with row standardization. Red and blue bars denote high and low expression, respectively.

The contents of catechins were found to vary in shoots according to metabonomic analysis, and ‘flavonoid biosynthesis’ was enriched in roots according to the KEGG analysis (Fig. 4). To fully understand the dynamic changes in gene expression in catechin metabolism under low-Mg^2+^ treatment, 42 unigenes encoding 12 enzymes were identified in the catechin biosynthesis pathway (Fig. 7 and Supplementary Data Table S5). Among them, cinnamate 4-hydroxylase (C4H) and anthocyanidin reductase (ANR) only corresponded to one gene, while other enzymes were found to belong to multiple gene families. In the conversion reaction from l-phenylalanine to naringenin, the expression levels of *PAL* (phenylalanine ammonia-lyase), *4CL* (4-coumarate-CoA ligase), *CHS* (chalcone synthase) and *CHI* (chalcone isomerase) were almost downregulated by low-Mg^2+^ stress, while the expression level of *C4H* increased. Flavanone 3-dioxygenase (F3H), flavanone 3′-dioxygenase (F3′H) and flavonoid 3′5′-hydroxylase (F3′5′H) catalyzed the formation of dihydrokaempferol, dihydroquercetin, and dihydromyricetin, respectively. Among them, *F3′5′H* was found to show upregulation in both shoots and roots under limited Mg^2+^ concentrations. Dihydroflavonol reductase (DFR) was considered to be involved in the synthesis of leucoanthocyanidins, whose expression displayed a general downtrend in shoots and uptrend in roots. Anthocyanidin synthase (ANS) catalyzed the formation of anthocyanidins, and its expression level was positively regulated in both shoots and roots. Leucoanthocyanidin reductase (LAR), which catalyzed the synthesis of catechin (C) and gallocatechin (GC), was almost upregulated, whereas ANR participating in the synthesis of epicatechin (EC) and epigallocatechin (EGC) showed a general decrease in both shoots and roots. Nevertheless, epigallocatechin acyltransferase (ECGT), which was reported to catalyze the formation of epicatechin gallate (ECG) and epigallocatechin gallate (EGCG), was not identified based on the transcriptome data. Overall, these findings indicated that catechin metabolism might act as a defense in response to Mg^2+^ deficiency.

### Expression profiles of genes involved in Mg^2+^ transport in *C. sinensis* under Mg^2+^ deficiency

Mg^2+^ transport systems play a key role in Mg^2+^ homeostasis. Hence, the expression profiles of genes in Mg^2+^ transport systems (10 *CsMGT*s and 1 *CsMHX*) were analyzed in this study (Supplementary Data Table S6). *CsMGT2*, *CsMGT4*, *CsMGT5*, and *CsMGT10* were upregulated in both tea plant shoots and roots. Among them, *CsMGT5* showed preferential expression in roots and a high expression abundance. Furthermore, the expression pattern of *CsMHX* was induced in *C. sinensis* shoots, while the expression profiles of *CsMGT6* and *CsMGT7* were increased in *C. sinensis* roots, indicating that Mg^2+^ transport systems played a vital part in maintaining the regular physiological activity of tea plants under Mg^2+^-limited conditions.

### Screening of key genes in regulation of Mg^2+^ homeostasis in *C. sinensis*

**Figure 8:**
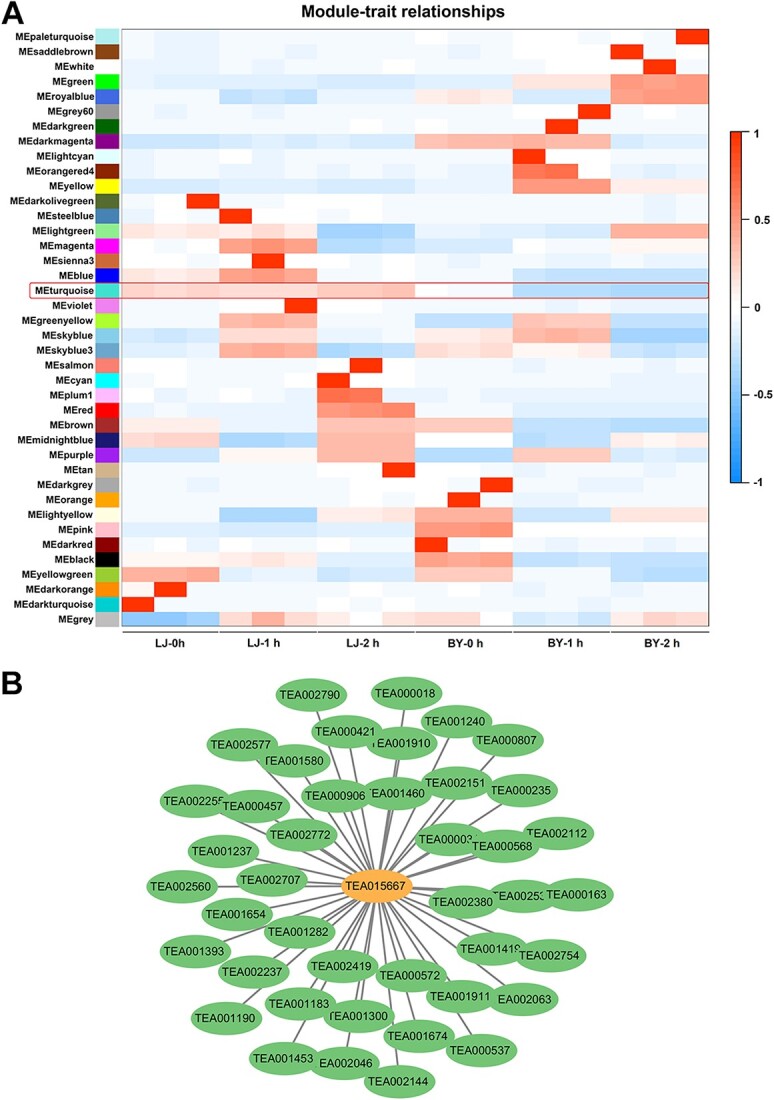
WGCNA of candidate genes that regulate the Mg^2+^ homeostasis in tea plants. **A** Correlations between modules and samples. The red box highlights the turquoise module. **B** Regulatory network constructed for the sample-associated module (turquoise module). The orange circle shows the prioritized causal gene (*CsMGT5*, TEA015667) for low-Mg^2+^ stress and green circles display its co-expressed genes in the turquoise module.

To further screen key genes that respond quickly to low-Mg^2+^ stress and maintain Mg^2+^ homeostasis in *C. sinensis*, annual cuttings of ‘Longjing 43’ and ‘Baiye 1’ were exposed to low-Mg^2+^ stress, and their young shoots were sampled at 0, 1, and 2 h for transcriptome sequencing. A total of 39 co-expression modules were constructed using a weighted gene co-expression network analysis (WGCNA) algorithm based on the transcriptome data of *C. sinensis* (‘Longjing 43’ and ‘Baiye 1) under short-term low-Mg^2+^ treatment. (Fig. 8A). The size of these gene modules ranged from 7071 eigengenes (turquoise module) to 38 eigengenes (orange red 4 module). The module–sample relationships were analyzed to identify the specific modules related to different tea varieties (i.e. ‘Longjing 43’ and ‘Baiye 1’). Notably, the turquoise module was positively correlated with ‘Longjing 43’ but negatively correlated with ‘Baiye 1’ (Fig. 8A). Therefore, we target the turquoise module as a critical module. Generally, edge weight reflects the strength of the communication between two genes in the WGCNA network, and the sum of weights on all edges of a node was used to define the connectivity level. Hub genes are considered as nodes with high connectivity, which are likely to play a key role in the module. *CsMGT5*, which was related to Mg^2+^ transport and possessed relatively higher connectivity, was identified as a hub gene from the turquoise module. Based on the weight value, genes co-expressed with *CsMGT5* were selected from the turquoise module and the co-expressed network was visualized by Cytoscape software (Fig. 8B). Furthermore, genes co-expressed with *CsMGT5* were subjected to KEGG pathway analyses. The co-expressed genes are involved in the biosynthesis of many metabolites, including amino acids, zeatin, phenylpropanoid, anthocyanin, starch, and sucrose. Besides, some genes respond to ions or participate in ion transport, playing a role in maintaining ion homeostasis. Moreover, some genes are annotated on important metabolic pathways, including carbon metabolism, pyruvate metabolism, purine metabolism, pyrimidine metabolism, and nicotinate and nicotinamide metabolism (Supplementary Data Table S7). Overall, these findings indicate that CsMGT5 may be a key regulator that responds quickly to low-Mg^2+^ stress and maintains Mg^2+^ homeostasis in *C. sinensis*.

### Suppression of *CsMGT5* reduced chlorophyll and carotenoid accumulation in *C. sinensis*

**Figure 9:**
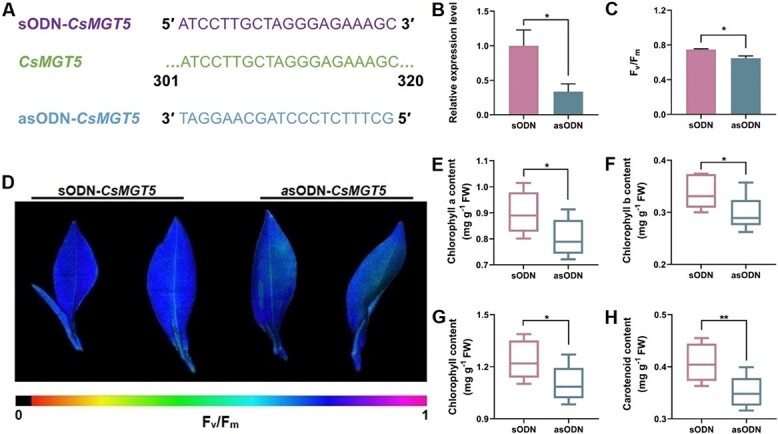
Functional characterization of *CsMGT5* in *C. sinensis*. **A** Primers used for silencing *CsMGT5* in asODN assay. **B** Expression profiles of *CsMGT5* in *C. sinensis* shoots after treatment with sODN-*CsMGT5* and asODN-*CsMGT5*. **C**  *F*_v_/*F*_m_ values. **D** Phenotypes of *CsMGT5*-silenced *C. sinensis* shoots. **E**–**H** Contents of chlorophyll a (**E**), chlorophyll b (**F**), chlorophyll (**G**), and carotenoid (**H**) in *C. sinensis* shoots treated with sODN-*CsMGT5* and asODN-*CsMGT5*. Error bars represent standard errors of three biological replicates. *P < 0.05; **P < 0.01 (Student’s *t*-test).

To explore the potential function of *CsMGT5* in *C. sinensis*, the asODN gene-specific interference strategy was employed to silence the expression of *CsMGT5* (Fig. 9A). Compared with sODN-*CsMGT5*, the expression of *CsMGT5* was significantly inhibited in *C. sinensis* shoots treated with asODN-*CsMGT5* (*P* < 0.05) (Fig. 9B). Further analysis indicated that *CsMGT5*-silenced shoots exhibited lower *F*_v_/*F*_m_ values than those of sODN-*CsMGT5* (*P* < 0.05) (Fig. 9C and D). Additionally, asODN-*CsMGT5* treatments decreased the contents of Chl a, Chl b, total Chl, and carotenoid in shoots (Fig. 9E–H). These findings suggested that *CsMGT5* might participate in pigment metabolism and photosystem II activity in *C. sinensis*.

### Heterologous expression of *CsMGT5* conferred resistance to Mg^2+^ limitation in transgenic *A. thaliana*

**Figure 10:**
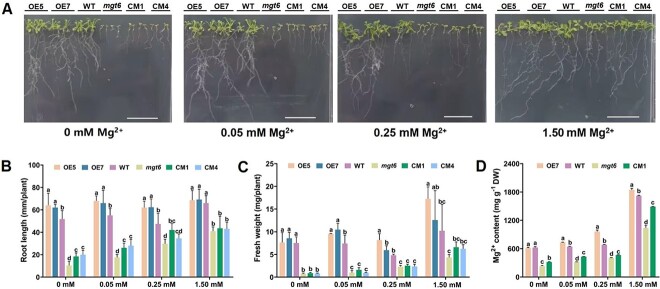
Assessment of low-Mg^2+^ tolerance of *CsMGT5*-transgenic *A. thaliana* plants subjected to low-Mg^2+^ treatments. **A**–**D** Phenotypes (**A**), root length (**B**), fresh weight (**C**) and Mg^2+^ content (**D**) in *CsMGT5*-overexpressing strains (OE5 and OE7), WT, *atmgt6* mutant (*mgt6*), and *atmgt6* complementary lines (CM1 and CM4) under different concentrations of Mg^2+^ (0, 0.05, 0.25, and 1.50 mM). Error bars represent standard errors of six biological replicates. Different letters indicate significant difference at *P* < 0.05. Scale bars = 2.5 cm.

To further clarify the function of *CsMGT5*, *CsMGT5* overexpression lines (OE5 and OE7) were generated on the basis of wild-type (WT) and *atmgt6* complementary strains (CM1 and CM4) were generated on the basis of the *atmgt6* mutant of *AtMGT6*, the homolog of *CsMGT5*. Under sufficient Mg^2+^ conditions (i.e. 1.50 mM), the phenotypes of WT, *mgt6*, and *CsMGT5* overexpression lines and *atmgt6* complementary lines had no significant difference, especially for root length and fresh weight (Fig. 10A–C). Although Mg^2+^ deficiency inhibited the growth of *A. thaliana* plants (Fig. 10A–C), the root lengths of *CsMGT5* overexpression lines and *atmgt6* complementary lines were longer than those of WT and *mgt6* under Mg^2+^-limited conditions (0, 0.05, and 0.25 mM), respectively (Fig. 10B). Moreover, there was no apparent difference in fresh weight between complementary lines and *mgt6* under Mg^2+^ deprivation (0 mM), but the fresh weight of *CsMGT5* overexpression lines was higher than that of WT when exposed to Mg^2+^ deficiency (0.05 and 0.25 mM) (*P* < 0.05) (Fig. 10C). Additionally, *CsMGT5* overexpression and complementation increased the contents of Mg^2+^ in transgenic *A. thaliana* compared with WT and *mgt6*, respectively (Fig. 10D). These findings implied that heterologous expression of *CsMGT5* maintained the growth of transgenic *A. thaliana* plants by increasing Mg^2+^ uptake and transport under Mg^2+^ deficiency and even starvation.

### *CsMGT5* overexpression increased the abundance of *AtAMT*s and amino acid contents in transgenic *A. thaliana*

**Figure 11:**
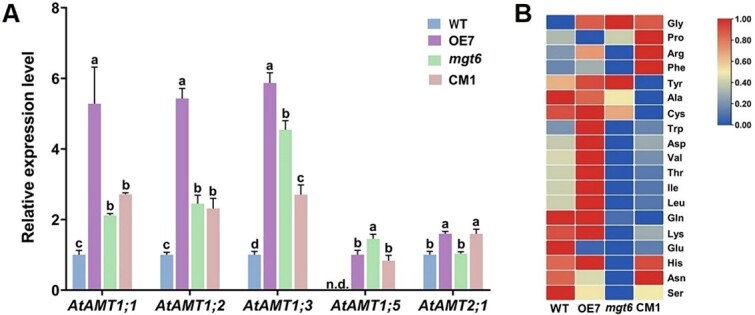
Effects of *CsMGT5* overexpression on expression of *AtAMT*s and contents of amino acids in *A. thaliana*. **A** Expression profiles of *AtAMT*s in *A. thaliana* roots. **B** Amino acid contents in *A. thaliana* leaves. OE7, *CsMGT5*-overexpressing line; *mgt6*: *atmgt6* mutant; CM1, *atmgt6* complementary line. Expression levels of *AtAMT1;1*, *AtAMT1;2*, *AtAMT1;3*, and *AtAMT2;1* in WT and *AtAMT1;5* in OE7 were set to 1.0. n.d. indicates the transcript was undetected. Different letters on the top of the error bars indicate significant difference at *P* < 0.05. The contents of each kind of amino acid in the heat map were analyzed with row standardization.

To explicit the molecular mechanism of *CsMGT5* in N metabolism, the expression profiles of ammonium transporters (AMTs) were measured in transgenic *Arabidopsis*. *AtAMT1;1*, *AtAMT1;2*, *AtAMT1;3*, and *AtAMT1;5* mediated NH_4_^+^ acquisition from soil solution; their expression levels in *CsMGT5* overexpression line roots were obviously higher compared with those in WT (*P* < 0.05) (Fig. 11A). Meanwhile, *AtAMT2;1*, participating in the process of root-to-shoot NH_4_^+^ translocation, was upregulated in *CsMGT5* overexpression lines and complementary lines (Fig. 11A). Further analysis of the amino acids showed that *CsMGT5* overexpression and complementation elevated the contents of total amino acids in transgenic *A. thaliana* compared with WT and *mgt6*, respectively (Fig. 11B and Supplementary Data Table S8). Among the amino acids, the contents of Arg, Asp, Leu, Ile, Thr, Trp, and Val of *CsMGT5* overexpression and complementation lines were obviously increased compared with WT and *mgt6*, respectively (Fig. 11B and Supplementary Data Table S8). Besides, *CsMGT5* overexpression lines improved the Gly content compared with WT (Fig. 11B and Supplementary Data Table S8). Furthermore, the content of Glu was decreased in *CsMGT5* overexpression lines compared with WT (Fig. 11B and Supplementary Data Table S8), so we speculated that CsMGT5 could promote the absorption of Mg^2+^ and induce the expression of *AtAMTs* to improve the uptake and translocation of NH_4_^+^, which might enhance N assimilation mediated by glutamine synthetase. Notably, we noticed that Pro increased obviously in CM1 (Fig. 11B and Supplementary Data Table S8), indicating that CsMGT5 may contribute to the enhancement of stress resistance by positively modulating the synthesis of proline in tea plants. Overall, this evidence indicates that *CsMGT5* could induce the expression of *AtAMT*s to promote the uptake and translocation of NH_4_^+^, thus increasing amino acid contents.

## Discussion

### Mg^2+^ deficiency disrupts sucrose and starch accumulation and chlorophyll metabolism in *C. sinensis*

Mg^2+^ is an indispensable nutrient in physiological and biochemical processes of plants [51]. Mg^2+^ deficiency hindered plant growth and development by disrupting key metabolic pathways, including photosynthesis, C assimilation, and N metabolism [26]. A typical symptom caused by Mg^2+^ deficiency is interveinal chlorosis of leaves, resulting from damage to the ultrastructure of chloroplasts [52]. This characteristic symptom appeared in coffee and banana under Mg^2+^ deficiency for 20 and 49 days, respectively [28, 29]. The *C. sinensis* cultivar ‘Longjing 43’ also exhibited the symptom when subjected to Mg^2+^ deficiency for 21 days [4]. In this study, tea plant cultivar ‘Zhongcha 108’ did not present obvious chlorosis after transition to low-Mg^2+^ conditions for 15 days (Fig. 1B), possibly due to the fact that lower Mg^2+^ concentrations did not cause leaf chlorosis within 15 days. Chlorosis induced by Mg^2+^ shortage is evident at a relatively late stage, while the accumulation of sucrose and starch in the source leaves is generally observed in the early Mg^2+^-limited period [52]. The accumulation of carbohydrates (i.e. sucrose and starch) was attributed to Mg^2+^ starvation inhibiting sucrose transporters responsible for loading sucrose in the phloem, finally resulting in an increase in the dry weight of shoots/roots [53]. Moreover, excessive accumulation of carbohydrates (i.e. sucrose and starch) repressed the expression of Chl a/b binding protein (CAB), causing a decrease in photosynthetic rate [54]. Besides, the unutilized light energy could produce ROS, further impairing the chloroplast membrane and affecting C assimilation [55]. Herein, KEGG analysis showed that multiple DEGs identified from tea plant shoots and roots were associated with the ‘starch and sucrose metabolism’ pathway, especially up to 53 DEGs in shoots (Fig. 4A). The dry weight of shoots/roots in tea plants showed a significant increase when treated with low Mg^2+^ (Supplementary Data Fig. S1), which is consistent with that in coffee, maize, and wheat [28, 56]. The photosynthetic characteristic parameters showed that the SPAD value decreased and *A* was inhibited by low-Mg^2+^ stress (Fig. 2B and E). In the Chl metabolism pathway, it was observed that low-Mg^2+^ conditions downregulated the expression of *CHLG* (Fig. 5), which catalyzed the formation of Chl a/b, resulting in the decline of Chl contents. Previous studies have clarified that Mg^2+^ deprivation affected the Chl a to Chl b ratio. For example, Mg^2+^-deficient mangoes have a higher ratio of Chl a to Chl b than normal plants, whereas the ratio declined in wheat leaves under Mg^2+^ limitations [57, 58]. In this study, the ratio of Chl a to Chl b declined apparently under low Mg^2+^ availability (Fig. 2F), implying that Mg^2+^ deprivation could lead to diverse impacts on the contents of Chl a and Chl b.

### Mg^2+^ deficiency inhibits amino acid accumulation in *C. sinensis*

Prior research generally confirmed that Mg^2+^ shortage influenced the content of metabolites. For instance, when exposed to Mg^2+^ deficiency for 8 days, 60 and 33 different metabolites, including organic acids, amino acids, and carbohydrates, were detected in soybean leaves and roots, respectively [59]. Besides, Mg^2+^ deficiency downregulated the biosynthesis of ubiquinones, terpene quinones, and various flavonoids in *Citrus* leaves [60]. In this study, we also found that the contents of multiple metabolites, which contained 19 amino acids, 10 flavonoids, 3 alkaloids, 6 catechins, and 10 organic acids, showed an alteration in tea plants after low-Mg^2+^ treatments (Fig. 3). It was reported that N deficiency downregulated the expression of *CsTS* (*theanine synthesis*) in *C. sinensis* roots, while the content of theanine increased in roots [17]. Adequate Mg^2+^ nutrition contributed to the biosynthesis of theanine in tea plant roots and its transport to shoots [61], but Mg^2+^ deficiency led to the accumulation of N and free amino acids in *C. sinensis* roots [47]. Intriguingly, we noticed that the contents of most amino acids in tea plant shoots and roots changed significantly in response to low-Mg^2+^ treatments, especially theanine, whose content decreased most in the shoots but increased obviously in the roots (Fig. 3A). Nevertheless, the expression patterns of *CsTS* in roots was inhibited by low-Mg^2+^ treatments (Fig. 6). Therefore, we speculated that the changes in theanine content may be attributed to the complex regulatory network of tea plants responding to low-Mg^2+^ stress. Firstly, Mg^2+^ starvation promoted the absorption of ammonia, which was due to the reduced competition of cations in the soil, contributing to the synthesis of amino acids [59]. Besides, Mg^2+^ deficiency restrained the transport of N from roots to leaves [47], thereby resulting in the differential accumulation of theanine in roots and shoots. Additionally, the expression levels of six amino acid transporters [*CsAAP2* (TEA009392.1), *CsAAP5* (TEA033139.1), *CsCAT6* (TEA031817.1), *CsCAT9* (TEA020444.1), *CsLHT1* (TEA024584.1), and *CsLHT12* (TEA021847.1)] were downregulated in roots (Supplementary Data Table S9), suggesting their synergistic role in tea plants under low-Mg^2+^ conditions. CsMYB6 was reported to serve as an activator of theanine biosynthesis by directly binding to and activating the promoter of *CsTS1*, while CsMYB73 is a transcription inhibitor of theanine biosynthesis by suppressing the expression of *CsGS1* and *CsGS2* [62, 63]. MYB9 and MYB49 were predicted to participate in regulating the biosynthesis of theanine [64]. Hence, we suspected that the differentially expressed TFs in this study may also be involved in modulating the biosynthesis and transportation of theanine under low Mg^2+^ availability. Finally, the transcription levels of multiple Mg^2+^ transporters were upregulated when exposed to low-Mg^2+^ environments, so we supposed that Mg^2+^ transporter systems might maintain normal physiological activity of tea plants by transporting Mg^2+^ under low-Mg^2+^ conditions (Supplementary Data Table S6). GABA is a crucial active ingredient in tea plants, which is regarded as a cross node of C and N metabolism, and participates in maintaining the balance of C and N nutrients [65]. GABA accumulated rapidly under various stress conditions, while a reduction in GABA content was found after 7 days of low-Mg^2+^ treatments (Fig. 3A), which was concordant with that of tea plants exposed to cold stress [66, 67]. Consequently, we conjectured that the decreased GABA content was due to the limitations of Mg^2+^ concentration and exposure time, which requires further investigation.

### Mg^2+^ deficiency disorders catechin biosynthesis in *C. sinensis*

Catechin is not only an essential secondary metabolite in *C. sinensis*, but also the main component of tea polyphenols [68]. The types and contents of catechins in *C. sinensis* could be impacted by development stages and organ tissues [68]. Up to now, six catechin monomers, C, GC, EC, EGC, ECG, and EGCG, have been detected in tea leaves, but only one catechin monomer (EC) exists in tea roots [69]. Moreover, the contents of catechins in *C. sinensis* shoots are the highest, followed by tender stems, and the lowest in roots [70]. Adverse environmental factors usually lead to the excessive production of ROS in plants, while the enhanced biosynthesis of catechin may promote ROS clearance [71]. In this study, except for GCG, the contents of most catechin monomers in shoots declined under low-Mg^2+^ stress (Fig. 3D). Hence, we assumed that not all catechin monomers were involved in the removal of ROS under Mg^2+^-limited conditions and GCG was conducive to adjustment of low-Mg^2+^ stress. Previous studies have unveiled that the expression of *ANR* exhibited a downward trend when exposed to drought, ABA, and GA_3_ treatments [72]. We found that low-Mg^2+^ stress downregulated the expression of *ANR* (Fig. 7), which might inhibit the generation of ester catechins. Moreover, the upregulation of *ANS* and downregulation of *ANR* might induce the accumulation of anthocyanins, which regulated the adaptability of *C. sinensis* to low-Mg^2+^ stress. Furthermore, the expression profile of *CsF3*′*5*′*H* was reported to be positively correlated with the contents of catechins [73], but this relationship was disturbed by low-Mg^2+^ treatment (Fig. 7).

### Circadian rhythm regulation and phytohormones may be associated with the adaptation of *C. sinensis* to low-Mg^2+^ stress

The circadian rhythm can not only regulate the growth of plants, but also modulate the response and adaptation to environmental stimuli [74]. Several key genes have been identified in regulation of the circadian rhythm, including *CCA1* (*Circadian clock associated 1*), *LHY* (*Late elongated hypocotyl*), *TOC1* (*Timing of CAB expression 1*), *PRR* (*Pseudo response regulator*), *GI* (*Gigantea*), and *ELF* (*Early flowering*) [75]. Moreover, abiotic stresses (i.e. low temperature, drought, and nutrition stresses) have been confirmed to disrupt the biological clock of plants [76]. When subjected to Mg^2+^ deprivation, *Arabidopsis* roots and leaves exhibited disturbances in the circadian oscillator [77]. In this study, 24 and 13 DEGs were annotated in the ‘circadian rhythm’ pathway in tea plant shoots and roots, respectively (Fig. 4). Notably, *PRR5* was downregulated by low-Mg^2+^ treatments, while *LHY* showed an opposite tendency (Supplementary Data Table S10), agreeing with earlier findings that *PRR5* could inhibit the expression of *LHY* [78]. Overexpression of *AtCCA1* conferred cold tolerance in transgenic *Arabidopsis* [79], so we deduced that the accumulation of *LHY*, the homologous gene of *CCA1*, might improve the adaptability of *C. sinensis* to low concentration of Mg^2+^. However, the molecular mechanisms underlying the effects of Mg^2+^ deficiency on the circadian oscillator and physiological output pathways in tea plants need to be further explored.

Phytohormones regulate abiotic stresses responses in *C. sinensis* [80]. It has been reported that the metabolism of endogenous plant hormones (IAA, ABA, GA_3_, and BR) might regulate the tea plant’s response to drought [9]. Low temperature promoted the biosynthesis of ABA, indicating that ABA might serve as a protector against freezing cold [81]. Exogenous application of IAA helped to alleviate the detrimental impacts of cadmium on tea seedlings [82]. Previous studies have revealed that Mg^2+^ deficiency induced the signal transduction of ABA and catalyzed the biosynthesis of ethylene to enhance tolerance in *Arabidopsis* [83, 84]. In this study, the numbers of DEGs enriched in ‘plant hormone signal transduction’ in *C. sinensis* roots were the highest (Fig. 4B). Among them, *ETR2* (*ethylene receptor 2*), which was the first element of the ethylene signal transduction pathway, presented an upward trend in the root under low-Mg^2+^ treatments (Supplementary Data Table S10). Moreover, low-Mg^2+^ stress induced the expression profiles of two auxin-responsive protein genes (*IAA1* and *IAA16*), which were the core factors of auxin signal transduction. However, the expression of *IAA18* and gibberellin receptor gene *GID1B* were downregulated (Supplementary Data Table S10). Hence, we speculated that phytohormones were associated with the adaptation of *C. sinensis* to low-Mg^2+^ stress.

### The Mg^2+^ transporter system is essential for Mg^2+^ homeostasis in *C. sinensis*

To maintain Mg^2+^ homeostasis, Mg^2+^ imbalance might trigger the defense mechanism in plants. It has been proved that the expression of *MGT*s could be upregulated by Mg^2+^ shortage in *Arabidopsis*, citrus, and maize [31, 35, 36]. In this study, we found that there was no significant change in the level of *A* when exposed to low-Mg^2+^ conditions for 7 days (Fig. 2B). Simultaneously, GO analysis also displayed that DEGs were enriched in ‘transporter activity’ (Supplementary Data Fig. S2). The expression of *CsMGT*s was upregulated by low-Mg^2+^ treatments (Supplementary Data Table S6), suggesting that Mg^2+^ transport systems might take the major responsibility for maintaining Mg^2+^ homeostasis under Mg^2+^ deficiency. Thus, we preliminarily inferred that the Mg^2+^ transport systems might function as a major role in Mg^2+^ homeostasis regulatory network under Mg^2+^ deficiency in tea plants. Some MGTs have been proved to regulate plant growth and development. Transgenic *Arabidopsis* silencing of *MGT6* showed growth retardation under low-Mg^2+^ stress, while AtMGT9-RNA inference plants displayed abortive pollen phenotype [41, 85]. Herein, *CsMGT5* not only responded to low-Mg^2+^ stress, but was also highly expressed in root tissues (Supplementary Data Table S6). Besides, a previous study has elucidated that CsMGT5 served as a high-affinity Mg^2+^ transporter, mediating the absorption and translocation of Mg^2+^ [48]. Therefore, we further speculated that CsMGT5 was essential for maintaining Mg^2+^ homeostasis in tea plants. To verify this hypothesis, an asODN interference gene-specific repression strategy was adopted to silence the expression of *CsMGT5* in shoots of *C. sinensis*. Surprisingly, we found that downregulation of *CsMGT5* reduced Chl and carotenoid contents (Fig. 9E–H), suggesting that CsMGT5 was associated with leaf color. However, the specific molecular mechanisms need further exploration. Furthermore, transgenic *Arabidopsis* systems, including *CsMGT5*-overexpressing lines and the *atmgt6* mutant, was employed to demonstrate the physiologic functions of *CsMGT5*. We observed that the growth of *atmgt6* was retarded severely, while the CM strain could compensate for the growth defects to some extent (Fig. 10A). Meanwhile, OE lines also elevated tolerance to low Mg^2+^. We noticed plants that had grown well were accompanied by a high Mg^2+^ content (Fig. 10D). Thus, we speculated that Mg^2+^ deprivation induced the expression of *CsMGT5*, which affected plant growth and development by accumulating Mg^2+^ content.

### CsMGT5 collaborates with ammonium transporter systems to elevate the contents of amino acids

In the *Arabidopsis* genome, six *AMT* genes were identified, named *AtAMT1;1*, *AtAMT1;2*, *AtAMT1;3*, *AtAMT1;4*, *AtAMT1;5*, and *AtAMT2;1* [86]. Among them, *AtAMT1;1*, *AtAMT1;2*, *AtAMT1;3*, and *AtAMT1;5* were mainly expressed in the root tissues, mediating NH_4_^+^ absorption from soil or culture medium [87]. *AtAMT1;4*, specifically expressed in pollen, was verified as a high-affinity transporter [88]. *AtAMT2;1* was reported to be a participant in the root-to-shoot translocation of NH_4_^+^ [89]. *AtAMT1;1* was induced in response to N starvation, and was also regulated by light duration, light intensity, and carbon [90]. *AtAMT1;3* was essential for N-deprived and NH_4_^+^ toxicity conditions [91]. In this study, *AtAMT1;1*, *AtAMT1;2*, and *AtAMT1;3* were obviously induced and amino acid contents in the *CsMGT5*-overexpression line (OE7) were higher than in WT (Fig. 11), suggesting that CsMGT5 enhanced the NH_4_^+^ transport activity of the AtAMT system, thus promoting amino acid contents. Proline accumulation acts in an adaptive role in the stress-resistance process [92]. Proline accumulation would protect membranes to augment freezing tolerance during cold acclimation and drought-tolerant plants contained higher levels of proline [93, 94]. Herein, we noticed that proline increased obviously in CM1 (Fig. 11B), indicating that CsMGT5 could enhance stress resistance by positively regulating the synthesis of proline in plants. However, the content of proline in OE did not show an increase compared with WT, so we speculate that this unexpected result may be due to the instability of the transgenic *A. thaliana* system. Tea plants show a preference for NH_4_^+^, which was absorbed by roots and transformed to amino acids [95]. Amino acids contribute to sensory properties [96]. For example, theanine is an important component in the umami taste of green tea [97]. Besides, amino acids serve as precursors to participate in the formation of tea aroma [98]. Therefore, amino acid contents are positively correlated with tea leaf quality. CsMGT5 collaborates with the AMT system to elevate the content of amino acids, suggesting that CsMGT5 plays a significant part in the quality of tea leaves.

### Conclusions

**Figure 12:**
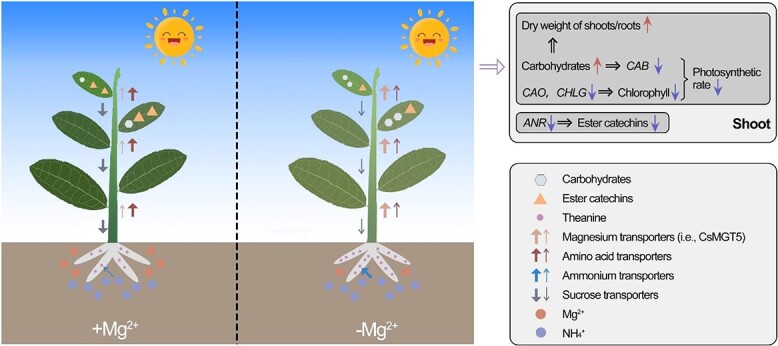
Summarized illustration of physiological and molecular insights underlying the Mg^2+^-deficiency response in *C. sinensis*. +Mg^2+^ and -Mg^2+^ indicate Mg^2+^-sufficient and Mg^2+^-deficient conditions, respectively. *CAB*, Chl a/b binding protein gene; *CAO*, chlorophyllide a oxygenase gene; *CHLG*, Chl a synthase gene; *ANR*, anthocyanidin reductase gene.

In summary, we combined the metabolome with the transcriptome to explore the effect of low Mg^2+^ on tea quality. Additionally, *CsMGT5*, screened by WGCNA, may be a key regulator that responds quickly to low-Mg^2+^ stress and maintain Mg^2+^ homeostasis in *C. sinensis*. Moreover, *CsMGT5* was functionally characterized by an asODN strategy and a transgenic *Arabidopsis* system. Furthermore, the novel finding that *CsMGT5* facilitates the AMT system to enhance amino acid accumulation offers a new insight for improving tea product quality. A working model is proposed to elucidate the molecular mechanisms in response to low-Mg^2+^ stress in *C. sinensis* (Fig. 12). Initially, Mg^2+^ deprivation suppressed the expression patterns of sucrose transporters, leading to the accumulation of carbohydrates in shoots, thereby increasing the ratio of shoot dry weight to root dry weight, indicating that Mg^2+^ shortage could affect the growth of *C. sinensis*. Meanwhile, the accumulation of carbohydrates inhibited the expression profile of *CAB*, which related to the photosynthetic rate. Besides, Mg^2+^ depletion inhibited the expression levels of *CAO* and *CHLG*, possibly leading to the reduction of Chl content, finally hindering the photosynthesis of tea plants. Additionally, the downregulation of *ANR* caused by Mg^2+^ limitations might reduce ester catechin contents. Moreover, the increase in theanine in roots may be attributed to complicated regulation networks, such as ion competition, the downregulation of amino acid transporters, and the upregulation of MGTs and AMTs. Furthermore, we also observed that Mg^2+^ depletion could disrupt the circadian rhythm, and that phytohormones may be involved in adaptation to Mg^2+^ imbalance. These preliminary findings demonstrated the impact of low Mg^2+^ on the quality of *C. sinensis*, providing theoretical guidance for nutritional management of tea plants.

## Materials and methods

### Plant materials and treatments

Annual tea plant cuttings [*C. sinensis* cv. ‘Zhongcha 108’ (ICR-23150)] were hydroponically pre-cultured in the South Lake Tea Garden of Huazhong Agricultural University as previously described [99]. For low-Mg^2+^ treatments, the tea plants, which had a uniform growth trend, were treated with 4.2 μM MgSO_4_, while other nutrients were consistent with the nutrient admixture [99]. Young shoots (one bud with two leaves) and roots were sampled after 7 days of treatments, and flash-frozen in liquid nitrogen and maintained at −80°C for metabolome and transcriptome analyses. Additionally, fresh samples were oven-dried at 103 ± 2°C to a constant weight to measure the dry weight of shoots and roots.

### Determination of photosynthetic physiological characteristic parameters

*E*, *A*, *C*_i_, and *g*_s_ in the second tea leaf were measured at a range of time points (0, 7, and 15 days) after low-Mg^2+^ treatments using a portable photosynthesis system (LI-6800, LI-COR, USA). The temperature, CO_2_ concentration, and light intensity of the leaf chamber were set at 25°C, 500 μmol s^−1^, and 1000 μmol m^−2^ s^−1^, respectively. Ten biological replicates were assessed for each treatment.

A Chl meter (SPAD-502, Konica Minolta, Japan) was utilized to determine the relative Chl contents in the second tea leaf, and each treatment consisted of 10 biological repeats. The concentrations of Chl a and Chl b in the second tea leaf were measured as previously [45], and each experiment was repeated four times.

### Metabolomic profiling analysis

The extraction process of metabolites in *C. sinensis* shoots and roots was performed as reported by Yu *et al*. [100] with moderate adjustments. Firstly, 0.15 g samples were ground fully using liquid nitrogen. Subsequently, 1.5 ml pre-cooled 75% (V/V) methanol solution containing 7.5 μg ml^−1^ D4-acetaminophen as internal standard were added to extract the metabolites. After 12 h of extraction under 4°C in the dark, the supernatant was obtained by centrifuging at 8000 g at 4°C for 5 min. Six replicates were prepared for each treatment. Furthermore, 100 μl supernatant from each treatment was mixed evenly as the quality control to assess the stability and repeatability of the metabolomic analysis. Metabolite profiling was conducted using a UHPLC system (Infinity 1290, Agilent Technologies, USA) coupled to a Q-TOF/MS instrument (Q-TOF 6520, Agilent Technologies, USA) and a Zorbax Eclipse Plus C18 reverse phase analytical column (1.8 μm, 2.1 mm × 150 mm; Agilent Technologies, USA). The specific conditions for chromatography and mass spectrometry were consistent with the method as previously published [101]. MassHunter Profinder (Version B.07.00, Agilent Technologies, USA) was applied to extract and align metabolic peaks. Finally, the following equation was employed to calculate the contents of detected compounds: relative content (μg g^−1^ DW) = peak area (compound)/peak area (internal standard) × 7.5 × 1.5/W (sample dry weight).

### RNA extraction, library construction, and RNA-seq

Total RNA was extracted using Trizol reagent (Invitrogen, CA, USA) based on the instructions. A Bioanalyzer 2100 and RNA 1000 Nano LabChip Kit (Agilent Technologies, CA, USA) were employed to analyze the quantity and purity of total RNA, and RIN number >7.0 was the qualified standard. Poly(A) RNA was purified and cleaved into small fragments. Then, the RNA fragments were reverse-transcribed to construct the final cDNA library. Finally, an Illumina HiSeq 4000 was applied to perform paired-end sequencing. Construction of the cDNA library and sequencing were entrusted to LC-Bio Technologies Co., Ltd (Hangzhou, China). For cost considerations, RNA-seq was performed with two independent biological replicates for each sample.

### Transcriptome assembly and functional annotation

Prior to assembly, raw reads were subjected to preprocessing to obtain clean reads, including removal of low-quality reads and reads with adaptor sequences. Hierarchical indexing for spliced alignment of transcripts [102] was used to map the clean reads to the *C. sinensis* cv. ‘Shuchazao’ genome [103]. GO was employed to analyze the molecular function, cellular component, and biological process of genes. The KEGG database helped to annotate the metabolic pathway. StringTie was applied to determine the expression level for mRNAs by calculating fragments per kilobase of exon model per million (FPKM) [104]. The value of |log_2_ratio| ≥ 1 and statistical significance (*P* < 0.05) were used to evaluate DEGs.

### Antisense oligonucleotide-mediated gene suppression

AsODNs for gene silencing were designed using Soligo software 2.2 with *CsMGT5* as an input [105]. Tender shoots (one bud with a leaf) were chosen and immersed in 20 μM asODN solution or 20 μM sODN solution. After incubation for 48 h, these shoots were sampled to determine gene expression levels, Chl and carotenoid contents, and *F*_v_/*F*_m_ values. A PAM-2000 Chl Fluorometer (Walz, Germany) was utilized to detect Chl fluorescence emission from the sample. The analyses were performed in triplicate.

### Functional verification of *CsMGT5* in transgenic *Arabidopsis*

The coding region of *CsMGT5* was cloned with primers (CsMGT5-F and CsMGT5-R) (Supplementary Data Table S11), followed by insertion into the plant expression vector pCAMBIA-2300-C-EGFP [106]. The fusion construct was introduced into *atmrs2-4* mutant (i.e. *atmgt6*) [107] and WT *Arabidopsis* plants based on the floral dip procedure [106]. Homozygous *T*_3_ transgenic lines were selected on Murashige and Skoog (MS) medium with 30 mg l^−1^ kanamycin.

The seeds were surface-sterilized and then cultured in agar-solidified MS medium containing 0, 0.05, 0.25, or 1.50 mM Mg^2+^. After cultivation in the growth chamber (22/20°C day/night; 16/8 h light/dark; relative humidity 75%; light intensity 150 μmol m^−2^ s^−1^) for 2 weeks, the seedlings were collected to measure the fresh weight and root length.

To detect the content of Mg^2+^, 0.1 g of dried sample was acid-digested with 4 ml of nitric acid and 1 ml of perchloric acid. The mixture was placed at room temperature overnight and then heated at 200°C until the digested material clarified. The solution was diluted to 50 ml with ultrapure water and filtered with a 0.22-μm membrane filter. Finally, inductively coupled plasma optical emission spectroscopy (Agilent 5110, CA, USA) was employed to determine the Mg^2+^ content at 279.6 nm wavelength. All treatments were performed with six biological replicates.

### Statistical analysis

Student’s *t*-test or one-way analysis of variance (ANOVA) was applied to determine differences, using IBM^®^ SPSS^®^ Statistics 25.0. Histograms and boxplot graphs were generated by GraphPad Prism 8.0.

## Supplementary Material

Web_Material_uhae152

## Data Availability

All relevant data in this study are provided in the article and its supplementary files.
