# Supplementary material for: Metabolome profiling and transcriptome analysis unveiling the crucial role of magnesium transport system for magnesium homeostasis in tea plants
Source: Hortic Res. 2024 Jun 3;11(7):uhae152. doi: 10.1093/hr/uhae152 (PMC11237192; doi:10.1093/hr/uhae152)
Supplement: Web_Material_uhae152 [file web_material_uhae152.zip › Supplemental Files.docx]

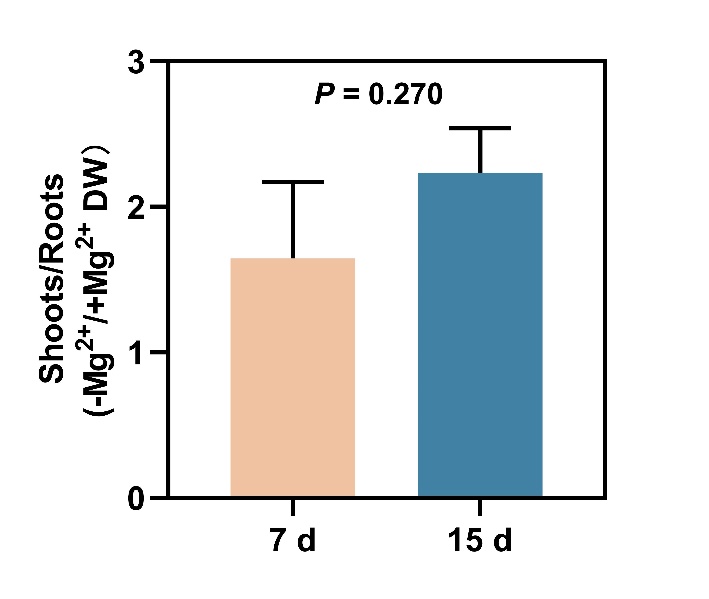


**Fig. S1.** Effect of Mg^2+^ deficiency on the growth of *C*. *sinensis*. The ratio of shoots to roots under Mg^2+^-sufficient conditions was set to 1.0, artificially.


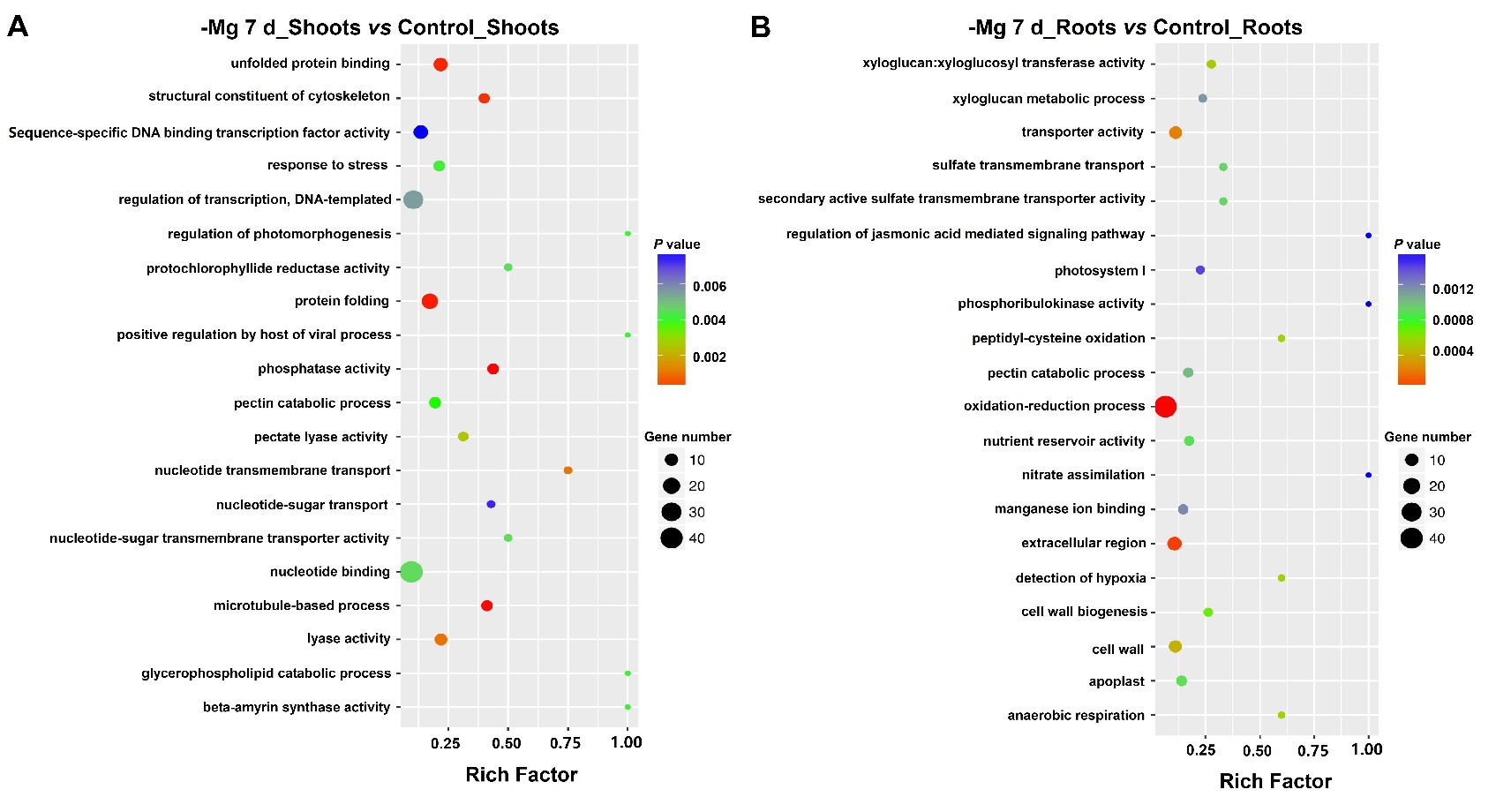


**Fig. S2.** The top 20 GO pathways enrichment of DEGs in comparison of (A) -Mg 7 d_Shoots *vs* Control_Shoots and (B) -Mg 7 d_Roots *vs* Control_Roots, respectively. The X-axis represents the enrichment factor and the Y-axis displays the name of GO term. The color of the circle refers to the *P* value and the size of the circle indicates the number of genes mapped to the pathway.

| **Table S1.** The dry weight of shoots and roots under Mg^2+^-sufficient (Control) and Mg^2+^ deficient (-Mg) conditions for 7 days and 15 days | | | | |
| --- | --- | --- | --- | --- |
|  | **Control 7 d** | **-Mg 7 d** | **Control 15 d** | **-Mg 15 d** |
| **Shoots** | 0.132 ± 0.024 | 0.224 ± 0.075 | 0.148 ± 0.027 | 0.421 ± 0.080 |
| **Roots** | 1.061 ± 0.115 | 1.116 ± 0.220 | 0.620 ± 0.153 | 0.780 ± 0.086 |
| Note: The data represent the mean ± SD (n = 3). | | | | |

| **Table S2.** The relative content of differential metabolites in shoots and roots of *C*. *sinensis* when treated with low-Mg^2+^ | | | | |
| --- | --- | --- | --- | --- |
| **Compounds** | **Control_Shoots** | **-Mg 7 d_Shoots** | **Control_Roots** | **-Mg 7 d_Roots** |
| L-Aspartate | 2.101 ± 0.150 | 2.091 ± 0.089 | 0.292 ± 0.100 | 0.457 ± 0.070 |
| L-Cysteine | 0.138 ± 0.020 | 0.151 ± 0.009 | - | - |
| L-Asparagine | 0.857 ± 0.037 | 0.865 ± 0.038 | 0.136 ± 0.021 | 0.407 ± 0.046 |
| L-Glutamate | 23.456 ± 0.855 | 11.842 ± 0.710 | 1.598 ± 0.010 | 1.917 ± 0.420 |
| L-Glutamine | 2.872 ± 0.117 | 1.08 ± 0.051 | 6.051 ± 0.060 | 6.735 ± 2.113 |
| L-Lysine | 2.880 ± 0.118 | 1.08 ± 0.050 | 6.059 ± 0.062 | 15.055 ± 0.288 |
| L-Threonine | 0.715 ± 0.061 | 0.238 ± 0.018 | 0.182 ± 0.002 | 0.194 ± 0.099 |
| L-Histidine | 2.713 ± 0.111 | 2.115 ± 0.066 | 2.127 ± 0.381 | 2.705 ± 0.938 |
| L-Proline | 0.486 ± 0.011 | 0.693 ± 0.026 | 1.645 ± 0.035 | 2.257 ± 0.922 |
| L-Valine | 0.684 ± 0.156 | 0.725 ± 0.045 | 0.192 ± 0.106 | 0.214 ± 0.134 |
| L-Theanine | 627.566 ± 142.290 | 466.846 ± 19.394 | 785.478 ± 43.234 | 1437.537 ± 47.839 |
| L-Tyrosine | - | - | 1.389 ± 0.010 | 1.525 ± 0.920 |
| L-Isoleucine | 0.869 ± 0.425 | 0.582 ± 0.320 | 3.281 ± 0.153 | 4.575 ± 0.135 |
| L-Leucine | 4.094 ± 2.164 | 2.198 ± 1.145 | - | - |
| L-Phenylalanine | - | - | 2.734 ± 0.007 | 2.959 ± 0.033 |
| L-Tryptophan | 9.110 ± 0.576 | 3.988 ± 1.818 | 3.570 ± 0.105 | 4.176 ± 0.001 |
| L-Methionin | - | - | 0.063 ±0.000 | 0.084 ± 0.156 |
| L-Serine | 0.098 ± 0.016 | 0.071 ± 0.003 | 0.014 ± 0.000 | 0.020 ± 0.002 |
| γ-aminobutyric acid | 1.114 ± 0.031 | 0.884 ± 0.032 | 0.200 ± 0.000 | 0.166 ± 0.004 |
| Myricetin 3-O-galactoside | 2.828 ± 0.032 | 3.832 ± 0.128 | 0.020 ± 0.000 | 0.029 ± 0.008 |
| Vitexin | 1.934 ± 0.043 | 1.123 ± 0.024 | 0.160 ± 0.000 | 0.021 ± 0.005 |
| Quercetin-7-O-β-D-glucopyranoside | 0.683 ± 0.041 | 0.758 ± 0.056 | - | - |
| Quercetin-3-o-rutinose | 1.302 ± 0.033 | 0.278 ± 0.024 | - | - |
| Vitexin-2″-o-rhamnoside | 6.473 ± 0.060 | 5.511 ± 0.071 | 0.399 ± 0.022 | 0.844 ± 0.156 |
| Quercetin-3-O-D-glucosyl]-(1-2)-L-rhamnoside | 0.448 ± 0.013 | 0.396 ± 0.021 | - | - |
| Isovitexin | 0.633 ± 0.032 | 0.419 ± 0.043 | 0.407 ± 0.000 | 0.033 ± 0.005 |
| Procyanidin B1 | 0.321 ± 0.164 | 0.181 ± 0.019 | - | - |
| Procyanidin B2 | 1.495 ± 0.031 | 0.519 ± 0.053 | - | - |
| Hyperoside | 0.537 ± 0.013 | 0.533 ± 0.021 | - | - |
| (-)-gallocatechin/GC | 7.634 ± 3.592 | 2.604 ± 0.371 | - | - |
| (-)-Epigallocatechin/EGC | 12.472 ± 5.701 | 4.193 ± 0.164 | - | - |
| Epigallocatechin gallate/EGCG | 99.549 ± 1.216 | 95.281 ± 1.381 | - | - |
| (-)-Gallocatechin gallate/GCG | 0.991 ± 0.124 | 1.600 ± 0.042 | - | - |
| (-)-Epicatechin gallate/ECG | 1.320 ± 0.022 | 1.173 ± 0.045 | - | - |
| Catechin gallate/CG | 35.958 ± 0.728 | 27.266 ± 0.604 | - | - |
| Theobromine | - | - | 1.889 ± 0.090 | 1.255 ± 0.590 |
| Theophylline | - | - | 0.333 ± 0.011 | 0.171 ± 0.021 |
| Caffeine | 570.037 ± 5.440 | 541.572 ± 10.732 | 1.446 ± 0.095 | 0.546 ± 0.028 |
| Shikimic acid | 392.771 ± 14.381 | 814.835 ± 48.932 | - | - |
| D-(-)-Quinic acid | 3.291 ± 0.133 | 2.717 ± 0.099 | 0.096 ± 0.024 | 0.146 ± 0.019 |
| Fumaric acid | 3.262 ± 0.066 | 3.186 ± 0.133 | 0.388 ± 0.042 | 0.940 ± 0.013 |
| Gallic acid | 0.303 ± 0.052 | 0.347 ± 0.065 | - | - |
| p-Coumaric acid | - | - | 0.050 ± 0.023 | 0.106 ± 0.018 |
| Sallcylic acid | - | - | 0.571 ± 0.021 | 0.787 ± 0.060 |
| Tartaric acid | 0.423 ± 0.023 | 0.340 ± 0.011 | 0.131 ± 0.000 | 0.119 ± 0.000 |
| α-Ketoglutaric acid | 2.863 ± 0.121 | 1.081 ± 0.058 | - | - |
| Citric Acid | 2.589 ± 0.124 | 2.715 ± 0.095 | - | - |
| Succinic acid | - | - | 1.487 ± 0.191 | 9.732 ± 1.514 |
| Note: "-" indicated the corresponding substance was undetectable. | | | | |

| **Table S3**. The FPKM values of key genes in biosynthesis pathway of chlorophyll in *C*. *sinensis* shoots under low-Mg^2+^ treatment | | | | | |
| --- | --- | --- | --- | --- | --- |
| **Enzyme name** | **Abbreviation** | **EC number** | **Gene ID** | **Control_Shoots** | **-Mg 7 d_Shoots** |
| Glutamyl-tRNA synthetase | GluRS | EC:6.1.1.17 | TEA002222.1 | 18.646 | 17.312 |
|  |  |  | TEA017365.1 | 16.795 | 18.475 |
| Glutamyl-tRNA reductase | HEMA | EC:1.2.1.70 | TEA032611.1 | 20.210 | 19.766 |
|  |  |  | TEA005844.1 | 20.756 | 9.137 |
|  |  |  | TEA026253.1 | 233.588 | 336.838 |
| Glutamate-1-semialdehyde aminotransferase | HEML | EC:5.4.3.8 | TEA031764.1 | 114.955 | 101.099 |
| Porphobilinogen synthase | HEMB | EC:4.2.1.24 | TEA012384.1 | 4.097 | 4.913 |
|  |  |  | TEA023791.1 | 58.551 | 80.797 |
| Hydroxymethylbilane synthase | HEMC | EC:2.5.1.61 | TEA022286.1 | 90.576 | 97.194 |
| Uroporphyrinogen-III synthase | HEMD | EC:4.2.1.75 | TEA028606.1 | 8.618 | 11.910 |
| Uroporphyrinogen decarboxylase | HEME | EC:4.1.1.37 | TEA016019.1 | 41.237 | 57.091 |
|  |  |  | TEA029169.1 | 11.161 | 9.808 |
|  |  |  | TEA009853.1 | 19.383 | 25.828 |
| Coproporphyrinogen III oxidase | HEMF | EC:1.3.3.3 | TEA015436.1 | 1.150 | 1.707 |
|  |  |  | TEA015415.1 | 41.475 | 35.018 |
| Menaquinone-dependent protoporphyrinogen oxidase | HEMG | EC:1.3.5.3 | TEA025819.1 | 17.559 | 18.762 |
|  |  |  | TEA004352.1 | 16.128 | 20.553 |
| Magnesium chelatase subunit H | CHLH | EC:6.6.1.1 | TEA021338.1 | 141.552 | 189.074 |
|  |  |  | TEA018733.1 | 113.957 | 83.940 |
|  |  |  | TEA032426.1 | 21.253 | 21.960 |
| Magnesium-protoporphyrin O-methyltransferase | CHLM | EC:2.1.1.11 | TEA025325.1 | 119.191 | 127.919 |
| Protochlorophyllide reductase | POR | EC:1.3.1.33 | TEA008264.1 | 42.533 | 100.803 |
|  |  |  | TEA029758.1 | 314.915 | 140.872 |
|  |  |  | TEA026812.1 | 4.830 | 12.023 |
|  |  |  | TEA014780.1 | 371.136 | 339.803 |
| Divinyl chlorophyllide a 8-vinyl-reductase | DVR | EC:1.3.1.75 | TEA006197.1 | 9.072 | 10.858 |
|  |  |  | TEA023688.1 | 32.156 | 48.752 |
| Chlorophyll a synthase | CHLG | EC:2.5.1.62 | TEA017076.1 | 42.113 | 38.523 |
| Chlorophyllide a oxygenase | CAO | EC:1.14.13.122 | TEA008213.1 | 298.062 | 295.799 |

| **Table S4.** The FPKM values of key genes in metabolic pathway of theanine in *C*. *sinensis* under low-Mg^2+^ treatment | | | | | | | |
| --- | --- | --- | --- | --- | --- | --- | --- |
| **Enzyme name** | **Abbreviation** | **EC number** | **Gene ID** | **Control** | **-Mg 7 d** | **Tissue** | **Annotation** |
| Alanine amino transferase | ALT | EC:2.6.1.2 | TEA016362.1 | 1.72 | 1.32 | root | Theanine biosynthesis |
|  |  |  | TEA023090.1 | 132.43 | 145.53 | root |  |
| Glutamine synthetase | GS | EC:6.3.1.2 | TEA015536.1 | 9.45 | 8.68 | root |  |
|  |  |  | TEA018359.1 | 4.33 | 5.28 | root |  |
|  |  |  | TEA032217.1 | 870.00 | 748.77 | root |  |
|  |  |  | TEA034053.1 | 10.23 | 7.92 | root |  |
|  |  |  | TEA032123.1 | 37.14 | 32.10 | root |  |
|  |  |  | TEA028194.1 | 45.21 | 45.74 | root |  |
|  |  |  | TEA034054.1 | 1.44 | 1.06 | root |  |
|  |  |  | TEA015580.1 | 207.42 | 215.44 | root |  |
| Glutamine-2-oxoglutarate synthetase | GOGAT | EC:1.4.1.13 | TEA003892.1 | 187.37 | 177.36 | root |  |
|  |  |  | TEA011569.1 | 185.73 | 169.70 | root |  |
| Glutamate dehydrogenase | GDH | EC:1.4.1.3 | TEA034006.1 | 0.78 | 0.95 | root |  |
|  |  |  | TEA034004.1 | 34.42 | 28.39 | root |  |
|  |  |  | TEA034047.1 | 1.42 | 0.51 | root |  |
|  |  |  | TEA006665.1 | 7.62 | 8.99 | root |  |
|  |  |  | TEA031206.1 | 37.72 | 11.29 | root |  |
| Theanine synthetase | TS | EC:6.3.1.6 | TEA015198.1 | 1193.59 | 929.57 | root |  |
| Pyridoxine biosynthesis 2 | PDX2 | EC:4.3.3.6 | TEA001280.1 | 23.35 | 23.38 | shoot | Theanine degradation |

| **Table S5.** The FPKM values of key genes in biosynthesis pathway of catechins in *C*. *sinensis* shoots and roots under low-Mg^2+^ treatment | | | | | | | | |
| --- | --- | --- | --- | --- | --- | --- | --- | --- |
| **Enzyme name** | **Abbreviation** | **EC number** | **Gene ID** | **Control_Shoots** | **-Mg 7 d_Shoots** | **Control_Roots** | **-Mg 7 d_Roots** |  |
| Phenylalanine ammonia-lyase | PAL | EC:4.3.1.24 | TEA034008.1 | 40.58 | 39.90 | 144.13 | 128.68 |  |
|  |  |  | TEA003137.1 | 10.83 | 7.21 | 46.04 | 48.54 |  |
|  |  |  | TEA024587.1 | 206.78 | 112.51 | 120.05 | 82.35 |  |
|  |  |  | TEA023243.1 | 2.09 | 2.69 | 0.36 | 0.24 |  |
|  |  |  | TEA014056.1 | 95.31 | 108.60 | 172.55 | 162.73 |  |
| Cinnamate 4- hydroxylase | C4H | EC:1.14.13.11 | TEA014864.1 | 155.28 | 253.26 | 954.96 | 742.14 |  |
| 4-coumarate-CoA ligase | 4CL | EC:6.2.1.12 | TEA027829.1 | 16.43 | 18.96 | 109.59 | 124.07 |  |
|  |  |  | TEA025906.1 | 40.24 | 42.74 | 123.38 | 145.90 |  |
|  |  |  | TEA034012.1 | 222.55 | 179.48 | 63.25 | 65.74 |  |
|  |  |  | TEA022274.1 | 1.61 | 0.97 | 3.25 | 3.31 |  |
|  |  |  | TEA019525.1 | 6.08 | 5.65 | 9.02 | 7.10 |  |
|  |  |  | TEA002100.1;TEA002062.1 | 18.93 | 14.73 | 17.06 | 15.50 |  |
|  |  |  | TEA009431.1 | 37.64 | 48.25 | 37.12 | 32.80 |  |
| Chalcone synthase | CHS | EC:2.3.1.74 | TEA023333.1;TEA023331.1 | 175.66 | 167.04 | 27.24 | 20.26 |  |
|  |  |  | TEA018665.1 | 23.12 | 18.97 | 0.19 | 1.30 |  |
|  |  |  | TEA023340.1 | 157.33 | 182.01 | 31.76 | 30.88 |  |
| Chalcone isomerase | CHI | EC:5.5.1.6 | TEA034003.1 | 112.03 | 78.15 | 43.56 | 49.43 |  |
|  |  |  | TEA018689.1 | 10.44 | 12.00 | 2.56 | 2.39 |  |
|  |  |  | TEA013101.1 | 57.75 | 55.13 | 43.03 | 43.47 |  |
|  |  |  | TEA033023.1 | 16.00 | 13.89 | 19.29 | 15.55 |  |
| Flavanone 3-dioxygenase | F3H | EC:1.14.11.9 | TEA034016.1 | 3.17 | 4.90 | 11.58 | 8.91 |  |
|  |  |  | TEA023790.1 | 657.29 | 560.33 | 67.17 | 51.17 |  |
| Flavonoid 3’5’-hydroxylase | F3′5′H | EC:1.14.13.88 | TEA026296.1;TEA026294.1 | 1.32 | 5.06 | 1.85 | 2.10 |  |
|  |  |  | TEA013315.1 | 289.63 | 527.72 | 3.55 | 3.87 |  |
| Flavanone 3’-dioxygenase | F3′H | EC:1.14.13.21 | TEA014658.1 | 7.12 | 0.00 | 0.27 | 0.21 |  |
|  |  |  | TEA016718.1;TEA016716.1 | 19.54 | 48.78 | 1.11 | 1.00 |  |
|  |  |  | TEA006847.1 | 254.19 | 168.44 | 82.62 | 106.85 |  |
| Dihydroflavonol reductase | DFR | EC:1.1.1.219 | TEA031287.1 | 2.36 | 2.65 | 18.01 | 9.82 |  |
|  |  |  | TEA023829.1 | 10.72 | 1.31 | 26.04 | 11.95 |  |
|  |  |  | TEA007645.1;TEA007646.1 | 2.78 | 1.63 | 1.44 | 1.62 |  |
|  |  |  | TEA024758.1 | 14.24 | 12.24 | 70.63 | 84.51 |  |
|  |  |  | TEA032730.1 | 66.25 | 54.31 | 104.23 | 113.14 |  |
|  |  |  | TEA022775.1 | 0.55 | 3.14 | 2.52 | 2.99 |  |
| Anthocyanidin synthase | ANS | EC:1.14.11.19 | TEA015769.1 | 1.34 | 3.38 | 1.55 | 6.78 |  |
| Anthocyanidin reductase | ANR | EC:1.3.1.112 | TEA005514.1 | 2.67 | 3.18 | 3.60 | 3.90 |  |
|  |  |  | TEA003752.1 | 2.60 | 2.46 | 4.10 | 3.71 |  |
|  |  |  | TEA009266.1 | 449.42 | 337.70 | 15.09 | 13.57 |  |
| Leucoanthocyanidin reductase | LAR | EC:1.3.1.77 | TEA027426.1 | 2.12 | 2.03 | 60.56 | 47.26 |  |
|  |  |  | TEA027538.1 | 299.02 | 278.90 | 320.42 | 486.61 |  |
|  |  |  | TEA029854.1 | 37.27 | 43.97 | 86.20 | 90.75 |  |
|  |  |  | TEA021535.1 | 5.53 | 7.83 | 9.10 | 14.04 |  |
|  |  |  | TEA027582.1 | 1031.70 | 1039.44 | 17.58 | 15.05 |  |

| **Table S6.** The FPKM values of *CsMGT*s and *CsMHX* in *C*. *sinensis* shoots and roots under Mg^2+^ deficiency | | | | | |
| --- | --- | --- | --- | --- | --- |
| **Gene ID** | **Gene name** | **FPKM.-Mg 7 d_Shoots** | **FPKM.Control_Shoots** | **FPKM.-Mg 7 d_Roots** | **FPKM.Control_Roots** |
| TEA005281.1 | *CsMGT1* | 8.99 | 9.11 | 2.14 | 2.56 |
| TEA011699.1 | *CsMGT2* | 7.30 | 7.04 | 7.15 | 5.75 |
| TEA015601.1 | *CsMGT3* | 6.68 | 8.93 | 11.94 | 12.95 |
| TEA030891.1 | *CsMGT4* | 14.98 | 13.57 | 12.90 | 11.07 |
| TEA015667.1 | *CsMGT5* | 1.80 | 1.29 | 38.08 | 36.85 |
| TEA010637.1 | *CsMGT6* | 14.51 | 15.51 | 17.42 | 13.66 |
| TEA016569.1 | *CsMGT7* | 4.88 | 5.25 | 1.72 | 1.50 |
| TEA011435.1 | *CsMGT8* | 0.39 | 0.36 | 39.07 | 43.67 |
| TEA009036.1 | *CsMGT9* | 27.45 | 28.95 | 18.65 | 20.25 |
| TEA005527.1 | *CsMGT10* | 73.02 | 59.97 | 5.24 | 4.43 |
| TEA006091.1 | *CsMHX* | 3.66 | 3.20 | 4.64 | 4.77 |

| **Table S7.** Annotation of genes co-expressed with *CsMGT5* | | |
| --- | --- | --- |
| **Gene ID** | **KEGG** | **Description** |
| TEA002772 | NA | hypothetical protein |
| TEA000031 | ko03013 (RNA transport) | importin subunit beta-1 |
| TEA001183 | NA | conserved oligomeric Golgi complex subunit 1 |
| TEA002046 | ko00230 (Purine metabolism); ko00670 (One carbon pool by folate) | phosphoribosylglycinamide formyltransferase, chloroplastic |
| TEA000568 | ko01200 (Carbon metabolism); ko00620 (Pyruvate metabolism); ko00710 (Carbon fixation in photosynthetic organisms) | phosphoenolpyruvate carboxylase 4 |
| TEA000906 | ko03050 (Proteasome) | 26S proteasome non-ATPase regulatory subunit 2 homolog A |
| TEA002754 | NA | hypothetical protein |
| TEA002151 | ko00230 (Purine metabolism) | allantoinase |
| TEA002790 | NA | annexin D4-like |
| TEA001393 | ko04626 (Plant-pathogen interaction) | receptor-like protein kinase FERONIA |
| TEA000018 | ko04141(Protein processing in endoplasmic reticulum) | chaperone protein dnaJ 1, mitochondrial |
| TEA002531 | NA | Aluminum sensitive 3 |
| TEA000572 | NA | NA |
| TEA002112 | ko04626 (Plant-pathogen interaction) | POPTRDRAFT_1092486 |
| TEA000807 | ko01200 (Carbon metabolism); ko01210 (2-Oxocarboxylic acid metabolism); ko01230 (Biosynthesis of amino acids); ko00020 (Citrate cycle (TCA cycle)) | SEC1 family transport protein SLY1-like |
| TEA001282 | NA | NA |
| TEA000163 | NA | NA |
| TEA002063 | ko00230 (Purine metabolism); ko00240 (Pyrimidine metabolism); ko00760 (Nicotinate and nicotinamide metabolism) | 5'-nucleotidase SurE |
| TEA001910 | ko03013 (RNA transport) | hypothetical protein |
| TEA002577 | NA | NA |
| TEA001190 | NA | NA |
| TEA002380 | NA | SORBIDRAFT_01g019720, Sb01g019720 |
| TEA002560 | NA | NA |
| TEA002419 | ko00908 (Zeatin biosynthesis) | SORBIDRAFT_01g001220, Sb01g001220 |
| TEA002255 | NA | endoplasmic reticulum-Golgi intermediate compartment protein 3-like |
| TEA001580 | ko04141(Protein processing in endoplasmic reticulum) | probable E3 ubiquitin ligase SUD1 |
| TEA000457 | NA | NA |
| TEA001300 | NA | uncharacterized LOC102585546 |
| TEA002707 | ko00500 (Starch and sucrose metabolism); ko00460 (Cyanoamino acid metabolism); ko00940 (Phenylpropanoid biosynthesis) | beta-glucosidase 12-like |
| TEA001453 | NA | uncharacterized LOC103718487 |
| TEA002144 | NA | NA |
| TEA001419 | NA | KH domain-containing protein HEN4 |
| TEA001460 | NA | NA |
| TEA001237 | NA | mitochondrial import inner membrane translocase subunit TIM50-like |
| TEA001654 | NA | potassium transporter 5-like |
| TEA001911 | NA | hypothetical protein |
| TEA000537 | NA | translation initiation factor IF-2, chloroplastic |
| TEA001674 | ko00460 (Cyanoamino acid metabolism) | putative serine carboxypeptidase-like 23 |
| TEA002237 | NA | cax1 |
| TEA001240 | ko00942 (Anthocyanin biosynthesis) | soyasaponin III rhamnosyltransferase-like |
| TEA000421 | ko04144 (Endocytosis) | delta(3,5)-delta(2,4)-dienoyl-CoA isomerase, peroxisomal-like |
| TEA000235 | ko00940 (Phenylpropanoid biosynthesis) | cinnamyl alcohol dehydrogenase 1 |

| **Table S8.** The contents of amino acids in *A*. *thaliana* | | | | |
| --- | --- | --- | --- | --- |
| **Compounds** | **WT (μg kg^-1^ DW)** | **OE7 (μg kg^-1^ DW)** | ***mgt6* (μg kg^-1^ DW)** | **CM1 (μg kg^-1^ DW)** |
| Gly | 0.003808811 | 0.007803356 | 0.008386295 | 0.007852196 |
| Ala | 0.00156784 | 0.001466793 | 0.001235382 | 0.000881379 |
| Arg | 0.39508542 | 0.781594056 | 0.226231417 | 1.004419582 |
| Asn | 0.007639165 | 0.005617918 | 0.003425236 | 0.008378193 |
| Asp | 0.025026501 | 0.048629865 | 0.010016256 | 0.020797582 |
| Cys | 0.005043354 | 0.0052985 | 0.004412824 | 0.002554796 |
| Glu | 0.132928629 | 0.095474093 | 0.093612157 | 0.098886748 |
| Gln | 0.084355547 | 0.084469874 | 0.069043878 | 0.067748358 |
| His | 0.015298999 | 0.016397707 | 0.009341735 | 0.015835671 |
| Ile | 0.004893969 | 0.007262118 | 0.003254198 | 0.003684017 |
| Leu | 0.004893969 | 0.007262118 | 0.003254198 | 0.003684017 |
| Lys | 0.077828865 | 0.079251963 | 0.06409582 | 0.068491522 |
| Phe | 0.027331679 | 0.028346329 | 0.026118549 | 0.033643143 |
| Pro | 0.965339113 | 0.859796536 | 0.989325402 | 1.178945376 |
| Ser | 0.05048231 | 0.047308982 | 0.043921097 | 0.047239673 |
| Thr | 0.005464185 | 0.010304726 | 0.002098747 | 0.003079671 |
| Trp | 0.003395312 | 0.012982701 | 0.000866079 | 0.002739914 |
| Tyr | 0.003418292 | 0.003586896 | 0.003634538 | 0.003009634 |
| Val | 0.128415617 | 0.24518428 | 0.03928361 | 0.078592918 |

| **Table S9.** The FPKM values of key amino acid transporters when treated with low-Mg^2+^ | | | |
| --- | --- | --- | --- |
| **Gene name** | **Gene ID** | **Control_Roots** | **-Mg 7 d_Roots** |
| *CsAAP2* | TEA009392.1 | 98.98 | 66.30 |
| *CsAAP5* | TEA033139.1 | 30.22 | 25.92 |
| *CsCAT6* | TEA031817.1 | 8.48 | 1.72 |
| *CsCAT9* | TEA020444.1 | 36.80 | 31.04 |
| *CsLHT1* | TEA024584.1 | 91.12 | 75.49 |
| *CsLHT12* | TEA021847.1 | 9.28 | 5.70 |

| **Table S10.** The FPKM values of key genes related to circadian rhythm and plant hormone signal transduction | | | | | | | | | |
| --- | --- | --- | --- | --- | --- | --- | --- | --- | --- |
| **Gene ID** | **Description** | **Control** | **-Mg 7 d** | **Tissue** | **Fold change** | **Log2(FC)** | **Regulation** | **Significant** | **Annotation** |
| TEA005441.1  TEA005447.1 | two-component response regulator-like APRR5; glucose-6-phosphate isomerase 1, chloroplastic-like | 17.06 | 1.85 | shoot | 0.16 | -2.61 | down | yes | Circadian rhythm related genes |
| TEA033521.1 | two-component response regulator-like APRR5 | 261.80 | 6.32 | shoot | 0.02 | -5.37 | down | yes |  |
| TEA018025.1 | protein LHY | 8.08 | 18.02 | root | 2.09 | 1.06 | up | yes |  |
| TEA020178.1 | ethylene receptor 2 | 4.56 | 17.55 | shoot | 3.85 | 1.95 | up | yes | Plant hormone related genes |
| TEA027708.1 | auxin-responsive protein IAA1-like | 6.02 | 18.79 | shoot | 3.12 | 1.64 | up | yes |  |
| TEA024199.1 | auxin-responsive protein IAA16 | 0.57 | 1.43 | shoot | 2.51 | 1.33 | up | yes |  |
| TEA018479.1 | auxin-responsive protein IAA18 | 39.71 | 10.87 | shoot | 0.27 | -1.87 | down | yes |  |
| TEA031063.1 | gibberellin receptor GID1B | 21.33 | 10.63 | shoot | 0.50 | -1.00 | down | yes |  |

| **Table S11.** Primers used in this study. | | |
| --- | --- | --- |
| **Primer name** | **Sequences (5′-3′)** | **Annotation** |
| CsMGT5-F | ATGGGGAAGGGGCCGTTCTG | Clone of *CsMGT5* |
| CsMGT5-R | AGATCCAAGCAGCTTCTTCG |  |
| AtAMT1;1-qF | GGATGAGATGGCCGGTATGG | qRT-PCR for *AtAMT*s |
| AtAMT1;1-qR | CACGATACCAGAAGGAGAAGGAGATCG |  |
| AtAMT1;2-qF | CGGCAAAAGCTGAAGTCGGAGC |  |
| AtAMT1;2-qR | GCCACCATAGCTTCTTCGCTTT |  |
| AtAMT1;3-qF | GCGGTTAACACCACACTCTC |  |
| AtAMT1;3-qR | TTACGTTCCAGTGGCCTGAT |  |
| AtAMT1;5-qF | GCTATTGGGAGCACAACTGG |  |
| AtAMT1;5-qR | TGCTGCTCCGAGATCCTAAG |  |
| AtAMT2;1-qF | TATGCTCTTTGGGGAGATGG |  |
| AtAMT2;1-qR | TGACACCTCTAGCACCATGAAC |  |
| AtACTIN2-qF | CTCCCGCTATGTATGTCGCC | *A*. *thaliana* internal reference gene |
| AtACTIN2-qR | TTGGCACAGTGTGAGACACAC |  |
