# Supplementary figures and images for: Metabolome profiling and transcriptome analysis unveiling the crucial role of magnesium transport system for magnesium homeostasis in tea plants
Source: Hortic Res. 2024 Jun 3;11(7):uhae152. doi: 10.1093/hr/uhae152 (PMC11237192; doi:10.1093/hr/uhae152)

Fig. S1.

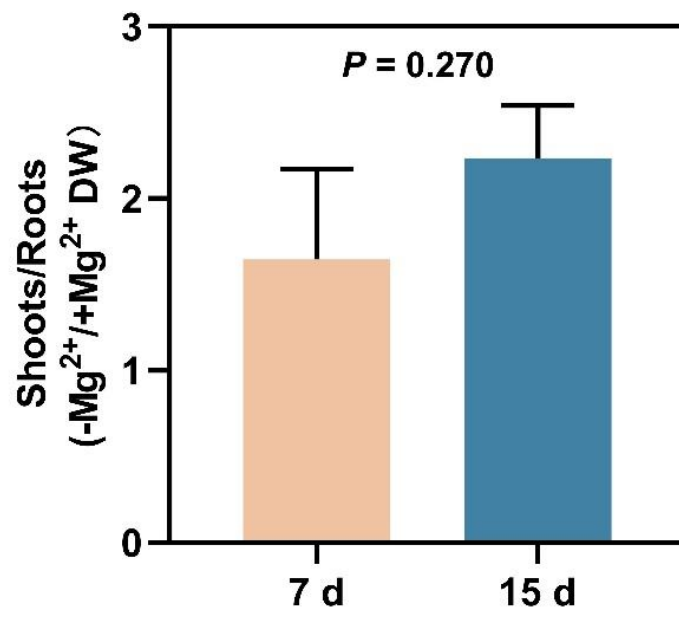

Fig. S2.

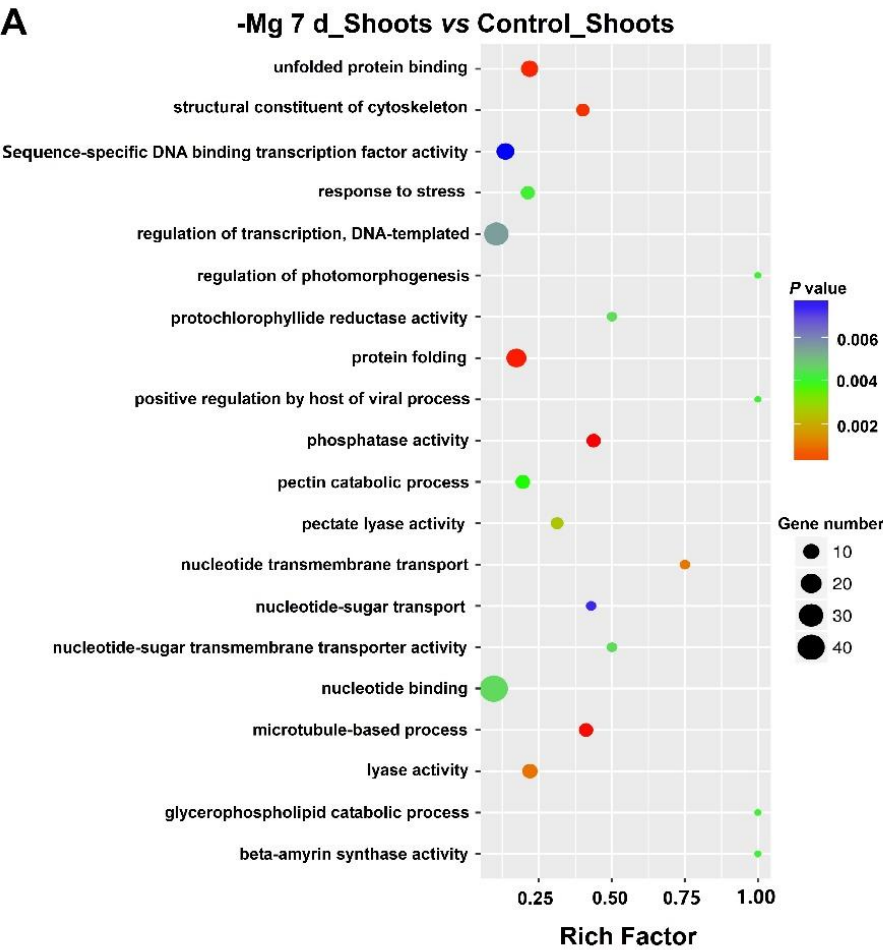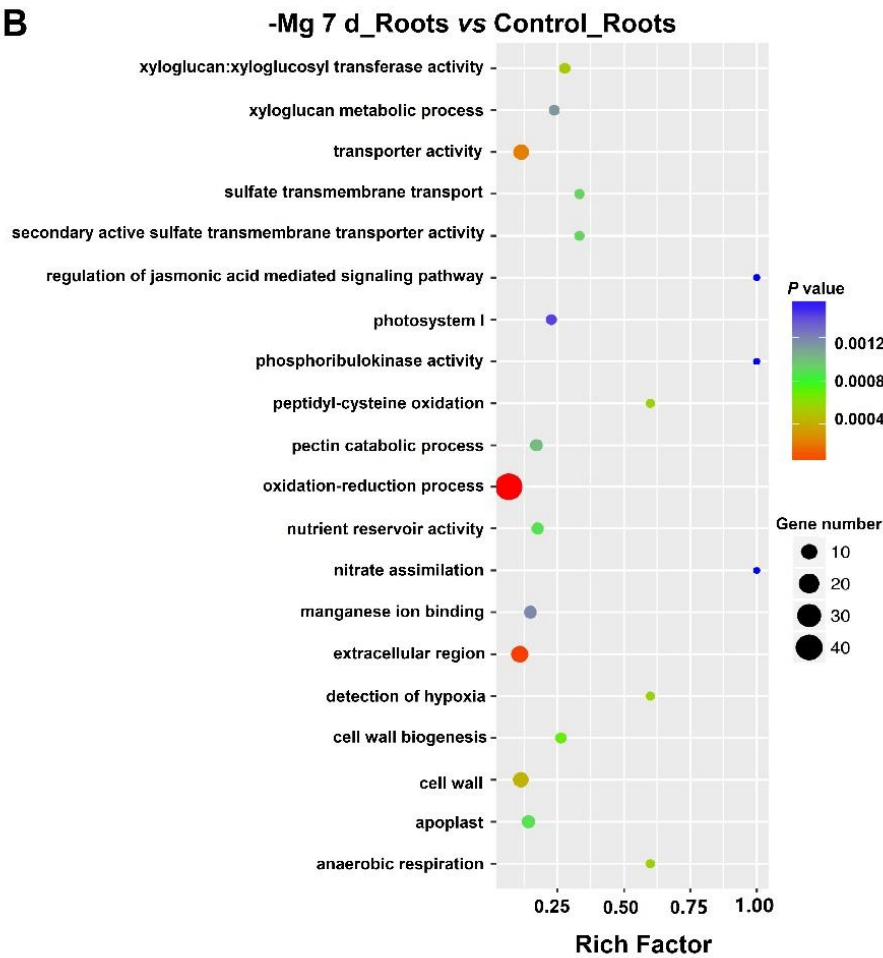

Supplement: Web_Material_uhae152 [file web_material_uhae152.zip › Supplementary Figures.pdf]
